# *Cinnamomum cassia* exhibits antileishmanial activity against *Leishmania donovani* infection *in vitro* and *in vivo*

**DOI:** 10.1371/journal.pntd.0007227

**Published:** 2019-05-09

**Authors:** Farhat Afrin, Garima Chouhan, Mohammad Islamuddin, Muzamil Y. Want, Hani A. Ozbak, Hassan A. Hemeg

**Affiliations:** 1 Department of Medical Laboratory Technology, Faculty of Applied Medical Sciences, Taibah University, Madinah, Kingdom of Saudi Arabia, Saudi Arabia; 2 Parasite Immunology Laboratory, Department of Biotechnology, Jamia Hamdard (Hamdard University), New Delhi, India; National Institutes of Health, UNITED STATES

## Abstract

**Background:**

There is a pressing need for drug discovery against visceral leishmaniasis, a life-threatening protozoal infection, as the available chemotherapy is antiquated and not bereft of side effects. Plants as alternate drug resources has rewarded mankind in the past and aimed in this direction, we investigated the antileishmanial potential of *Cinnamomum cassia*.

**Methodology:**

Dichloromethane, ethanolic and aqueous fractions of *C*. *cassia* bark, prepared by sequential extraction, were appraised for their anti-promastigote activity along with apoptosis-inducing potential. The most potent, *C*. *cassia* dichloromethane fraction (CBD) was evaluated for anti-amastigote efficacy in infected macrophages and nitric oxide (NO) production studied. The *in vivo* antileishmanial efficacy was assessed in *L*. *donovani* infected BALB/c mice and hamsters and various correlates of host protective immunity ascertained. Toxicity profile of CBD was investigated *in vitro* against peritoneal macrophages and *in vivo* via alterations in liver and kidney functions. The plant secondary metabolites present in CBD were identified by gas chromatography-mass spectroscopy (GC-MS).

**Principal findings:**

CBD displayed significant anti-promastigote activity with 50% inhibitory concentration (IC_50_) of 33.6 μg ml^-1^ that was mediated via apoptosis. This was evidenced by mitochondrial membrane depolarization, increased proportion of cells in sub-G_0_-G_1_ phase, ROS production, PS externalization and DNA fragmentation (TUNEL assay). CBD also inhibited intracellular amastigote proliferation (IC_50_ 14.06 μg ml^-1^) independent of NO production. The *in vivo* protection achieved was 80.91% (liver) and 82.92% (spleen) in mice and 75.61% (liver) and 78.93% (spleen) in hamsters indicating its profound therapeutic efficacy. CBD exhibited direct antileishmanial activity, as it did not specifically induce a T helper type (Th)-1-polarized mileu in cured hosts. This was evidenced by insignificant modulation of NO production, lymphoproliferation, DTH (delayed type hypersensitivity), serum IgG2a and IgG1 levels and production of Th2 cytokines (IL-4 and IL-10) along with restoration of pro-inflammatory Th1 cytokines (INF-γ, IL-12p70) to the normal range. CBD was devoid of any toxicity *in vitro* as well as *in vivo*. The chemical constituents, cinnamaldehyde and its derivatives present in CBD may have imparted the observed antileishmanial effect.

**Conclusions:**

Our study highlights the profound antileishmanial efficacy of *C*. *cassia* bark DCM fraction and merits its further exploration as a source of safe and effective antieishmanial compounds.

## Author summary

Leishmaniasis encompasses a broad spectrum of vector-borne neglected tropical diseases with significant worldwide health impact, ranging from the self-healing cutaneous lesions to stigmatizing and disfiguring skin ulcers (mucocutaneous), and the systemic visceral manisfestations (kala azar or visceral leishmaniasis, VL). A poverty-stricken disease, VL is fatal, if left untreated and resurfaces as post-kala azar dermal leishmaniasis after several years of apparent cure. Moreover, there is an upward trend in development of resistance to most of the currently available chemotherapeutic arsenal. Absence of vaccines, progressive emergence of HIV-*Leishmania* co-infection delineate the gravity of VL affliction. Natural products from medicinal plants have shown leishmanicidal effect, which in some cases, is potentiated by immunomodulation. Here, we elucidate the antileishmanial efficacy of *Cinnamomum cassia* bark fraction (CBD) with no adverse side effects. The parasites were eliminated by apoptosis *in vitro* while the protection *in vivo* was complemented by partial immunomodulation. CBD may be used in synergy with known or pipeline drugs for effective maintenance of VL. Our study represents an important step in the regional drive towards VL elimination.

## Introduction

Visceral leishmaniasis (VL) or kala-azar is the fatal form of leishmaniasis caused by *Leishmania donovani*, a digenetic parasite that preferentially invades the host liver, spleen and bone marrow macrophages. VL has colonized tropical and sub-tropical countries most of which are either under developed or developing where it clouts economically ailing population, causing an immense death toll of 20,000–40,000 each year [1]. The implications associated with VL are not only restricted to mortality; in fact, VL negatively impacts whole socio-economic structure of the affected society by producing physical and reproductive disabilities [2]. VL also occurs as a relapse infection in the form of post kala-azar dermal leishmaniasis (PKDL) in 5–10% of clinically recovered patients in India after 2–3 years of treatment and, in 50% of clinically cured patients in Sudan within 6 months of treatment duration. PKDL patients being highly infectious serve as putative reservoirs in between epidemic outbreaks posing a grave challenge against effective VL control [3]. Despite being a neglected tropical disease associated with poverty, VL has gathered significant attention recently due to its association with acquired immunodeficiency syndrome (AIDS). In its non-endemic foci, VL is a common co-infection with AIDS where up to 70% of adult leishmaniasis cases have been reported to be related with human immunodeficiency virus (HIV) [4].

More than 90% of the new VL cases arise in its endemic foci comprised by India, Bangladesh, Brazil, Sudan, South Sudan and Ethiopia where both death and drug resistance are at escalation [5]. About half of the global burden of VL is borne by India as per GBD statistics [6], in states of Bihar, and neighbouring districts of West Bengal, Uttar Pradesh and Jharkhand, with Bihar alone accounting for 90% of the cases [7]. Despite decades of research, no commercial vaccine is available against VL and chemotherapy is failing owing to emerging resistance, adverse side effects and cost [8]. Pentavalent antimonials, the 1^st^ line of drugs against leishmaniasis, are at back-foot with more than 60% of failure cases in Bihar, India, making Amphotericin B (AmB), as a drug of choice [9]. AmB exhibits high clinical efficiency but long treatment duration, parenteral administration and associated renal toxicity are hard to bear. Miltefosine, the first oral drug against the disease is teratogenic and its efficacy has been reduced by escalating drug resistance; and combination therapy of paromomycin and miltefosine is also not bereft of limitations [10].

The repercussions associated with VL are dreadful, chemotherapy is unsatisfactory and expansion of VL to its non-endemic areas has gathered sufficient attention for its immediate control. Unavailability of licensed antileishmanial vaccine despite few being in clinical or pre-clinical trial, and limited chemotherapy [8] has paved the way for drug discovery from alternate sources such as plants. Plant kingdom is endowed with unparallel molecular diversity that has aided mankind in discovery of many potent drugs including anti-protozoals [11]. Quinine derived from *Cinchona succirubra* till date remains an important antimalarial drug after forty decades of its discovery [12] and artemisinin derived from *Artemisia annua* has led to a paradigm shift in antimalarial research [13]. Exploration of plants for candidate antileishmanial compounds has been exhaustive and many plant extracts, fractions, and isolated plant secondary metabolites have shown significant leishmanicidal activities, some with potent immunomodulation [14]. Encouraged by these studies, and utilizing the benefit of leishmaniasis from drug repurposing or piggy-back chemotherapy [15], and further to open a possibility of VL elimination drive from the Indian sub-continent [16], we evaluated antileishmanial potential of *Cinnamomum cassia* (family, Lauraceae) which is commonly used in traditional Chinese medicine [17]. *C*. *cassia* has been reported to possess many pharmacological properties such as antibacterial [18], anticancer [19], antidiabetic [20], antifungal [21], neuroprotective [22] and prevents oxidative stress-related diseases [23]. To the best of our knowledge, this is the first report on *in vitro* and *in vivo* leishmanicidal potential of *C*. *cassia* against *L*. *donovani*.

## Materials and methods

All the chemicals used in study were either of analytical or molecular biology grade and procured from well-known standard companies. Fetal Bovine Serum (FBS) was purchased from GIBCO-BRL, 4-(2-hydroxyethyl)-1-piperazine-ethanesulfonic acid (HEPES) from Himedia Laboratories, Mumbai, India, in situ cell death detection kit, POD from Roche Inc., Switzerland, hexane and methanol from SD Fine-Chem Limited, Mumbai, India, copper sulphate, sulphuric acid, hydrochloric acid, sodium chloride, sodium dihydrogen phosphate, disodium hydrogen phosphate, hydrogen peroxide, citric acid, bovine serum albumin (BSA), Folin’s reagent, ammonium chloride, trypan blue, sodium nitrite, phosphoric acid, sulphanilamide, N-(1-napthyl) ethylenediamine dihyrochloride were procured from Merck. All other chemicals were obtained from Sigma-Aldrich unless mentioned otherwise.

### Ethics statement

For in vivo studies, the experimental protocols were approved by the Jamia Hamdard Animal Ethics Committee (JHAEC) (Ethical approval judgment number 499). JHAEC is registered under the Committee for the purpose of control and supervision of experiments on animals (CPCSEA). Female BALB/c mice aged 6–8 weeks (20–25) g and male Syrian golden hamsters 4–6 weeks of age were used for in vivo antileishmanial studies. All the animals were individually housed in standard size polycarbonate cages under standard conditions in the Central Animal House of Jamia Hamdard according to the internationally accepted principles. BALB/c mice were infected with *L*. *donovani* promastigotes (2.5×10^7^/animal) via tail vein while hamsters were infected intra-cardially. Mice were bled from retro-orbital plexus and hamsters by intra-cardiac puncture. The animals were anesthetized with isoflurane prior to carbon dioxide (CO_2_) euthanasia.

### Media and cell culture

*L*. *donovani* promastigotes were cultured in complete medium 199 (M199) (with 10% heat-inactivated FBS), pH 7.4, supplemented with penicillin G sodium (100 U ml^-1^), streptomycin sulfate (100 μg ml^-1^), HEPES (25 mM). The WHO reference strain of *L*. *donovani* (MHOM/IN/83/AG83) was obtained as a kind gift from Dr. Nahid Ali, Scientist, IICB, Kolkata. The *L*. *donovani* strain was maintained as amastigotes in BALB/c mice and promastigotes in culture as described previously [24].

To culture and perform studies with peritoneal macrophages, Roswell park memorial institute (RPMI)-1640 medium devoid of phenol red was used [24]. The media was supplemented with 100 μg ml^-1^ streptomycin sulfate, 100 U ml^-1^ penicillin G-sodium, 0.2% sodium bicarbonate, 25 mM HEPES. FBS (10%) was used as and when required according to experimental conditions. Also, for all *in vivo* experiments, splenic lymphocytes were isolated and cultured in phenol red free RPMI-1640 medium. All the cell cultures were maintained in a humidified atmosphere at 37 °C with 5% CO_2_.

### Plant material and extraction

*C*. *cassia* bark was purchased locally, and authenticated at NISCAIR, CSIR, New Delhi, by Dr. H.B. Singh (voucher no. NISCAIR/RHMD/Consult/-2010-11/1440/38). The extraction was performed as described previously by us [25] with few modifications. In brief, 100 g of powdered bark was immersed in 500 ml of dichloromethane (DCM) for 24 h, followed by four consecutive washes with half the volume of DCM at 24 h interval. This was followed by 5 washes each with ethanol and water in a sequential manner. The filtrate obtained at each step was passed through Whatman filter paper (No. 1) and pooled following concentration in a rotary evaporator at 35°C. The aqueous fraction was lyophilized. The dried fractions were kept at -20°C until used for bioassay.

### Assessment of anti-promastigote activity of CBD for determination of IC_50_ and cytocidal/ cytostatic mode of action

Bioactivity of *C*. *cassia* fractions was appraised by growth kinetics assay wherein stationary phase promastigotes (2×10^6^ cells ml^-1^) were cultured in the absence or presence of various test fractions (500 μg ml^-1^) at 22°C. Pentamidine (a known antileishmanial drug) served as positive control (500 μg ml^-1^) and dimethyl sulphoxide (DMSO, 0.25%) was taken as solvent control (maximum concentration used to solubilize the extracts) to ascertain any unspecific parasite death. Following treatment, culture aliquots from all the groups were taken at every 24 h for 7 days, erythrosin B (0.2%, 1:1) stained, and counted in a haemocytometer under phase contrast microscope (40X) to ascertain the cell density [25]. To obtain photomicrographs, treated and untreated cells were erythrosin B stained and observed under oil immersion at 100 X using a phase contrast microscope.

*C*. *cassia* fractions were examined for their cytocidal or cytostatic mode of action in a growth reversibility assay. Briefly, the treated or untreated parasites cultured as above were harvested after 7 days. The drug was withdrawn by washing the promastigotes twice (3000×g, 10 min, 4°C) with incomplete M199 (without FBS) and all the samples were re-incubated in fresh M199 (complete media, with 10% FBS) at 22°C. Post 96 h of incubation, culture aliquots were taken and cell density determined [25].

For determination of 50% promastigote growth inhibitory concentration (IC_50_), stationary phase parasites at a cell density of (2×10^6^ ml^-1^) were incubated either in the absence or presence of *C*. *cassia* bark DCM fraction (CBD) and pentamidine (500 μg ml^-1^) at serial two fold dilutions (500 to 3.90 μg ml^-1^) for 96 h at 22°C. The parasite survival was assessed by enumerating the live cells in a haemocytometer after erythrosin B staining. The cell density was determined as per the standard formula: [Cell count (in 16 squares) × dilution factor ×10^4^ = cell density (10^6^ ml^-1^)] and percent (%) viability was calculated as per the formula:

$$\%\;\;\text{Viability}=\frac{{\text{Average}\;\text{viable}\;\text{cell}\;\text{count}\;\text{per}\;\text{ml}}_{\left(\text{traated}\;\text{samples}\right)}}{{\text{Average}\;\text{viable}\;\text{cell}\;\text{count}\;\text{per}\;\text{ml}}_{\left(\text{parasite}\;\text{control}\right)}}\times 100$$

IC_50_, the concentration that inhibited the parasite growth by 50% was determined by graphical extrapolation [24].

### Analysis of apoptotic events in *L*. *donovani* promastigotes

#### Determination of Phosphatidylserine (PS) externalization

PS externalization as a marker of apoptosis was studied in treated and untreated *L*. *donovani* promastigotes as described previously [25]. Briefly, stationary phase promastigotes were adjusted to a cell density of 2×10^6^ cells ml^-1^ and subjected to treatment with IC_50_ concentration of CBD or pentamidine (taken at equivalent concentration). Post 72h, untreated and treated samples were processed and stained with Annexin-V and Propidium iodide (PI) according to instructions provided with Annexin-V FLUOS staining kit (Roche). The samples were acquired on BD LSR-II flow cytometer and 10, 000 events per sample were recorded followed by analysis using BD FACS DIVA software. Dot plots of FL-1 channel (Annexin-V) versus FL-2 (PI) were plotted and cell population in different quadrants was recorded.

#### Analysis of changes in mitochondrial membrane potential (Ψm)

Changes in Ψm were detected by staining with JC-1 (5,5',6,6'-tetrachloro-1,1',3,3'-tetraethylimidacarbocyanine iodide). JC-1 fluoresces differentially inside live and apoptotic cells. In live cells, it is retained inside mitochondria forming J-aggregates, whereas, in apoptotic cells it is expelled from the mitochondria, and resides in cytoplasm in monomeric state (J-monomers). Thus, ratio of J aggregates (red)/J monomers (green) is indicative of the relative Ψm of the cell [26]. Briefly, to determine Ψm in *L*. *donovani* promastigotes, the promastigotes (2×10^6^ ml^-1^) were treated in the absence or presence of IC_50_ of CBD (34 μg ml^-1^) for 72 h at 22°C in a BOD incubator. Pentamidine, was also tested in parallel at same concentration (34 μg ml^-1^). Post-treatment, the cells were harvested, washed twice with phosphate buffered saline (PBS) and stained with JC-1 (10 μg, 10 min, 37°C). After staining, the cells were again washed twice with PBS followed by acquisition in a BD LSR II flow cytometer. 10, 000 events were acquired and quadrant plots were created with the help of BD FACS DIVA software which were used to distinguish J-aggregates from J-monomers, the red (J-aggregates)/green (J-monomer) ratio was then calculated to ascertain the relative Ψm in untreated and treated samples [24].

#### Determination of reactive oxygen species (ROS) generation

ROS generation was monitored as described previously [25, 26]. *L*. *donovani* promastigotes (2×10^6^ cells ml^-1^) were treated with CBD and pentamidine (34 μg ml^-1^) or with medium alone for 72 h at 22°C, following which the cells were harvested and stained with 2′,7′- dichlorodihydrofluorescein-diacetate (H_2_DCFDA,10 μM, 15 min) which emits green fluorescence upon cleavage by ROS. Following incubation, the cells were acquired on a BD LSR II flow cytometer. Histograms were created and mean fluorescence intensity (MFI) of each sample was deduced from the histogram statistics.

#### Study of DNA fragmentation by terminal deoxynucleotidyltransferase (TdT) mediated dUTP (deoxyuridine triphosphate) nick end labeling (TUNEL) assay

During apoptosis, low and high molecular weight genomic DNA fragments can be detected by TUNEL assay, which uses TdT to incorporate labeled dUTP into free 3'-OH (hydroxyl) termini of the fragmented DNA. To detect *in situ* DNA fragmentation, *L*. *donovani* promastigotes (2×10^6^ ml^-1^) were incubated with CBD and pentamidine (34 μg ml^-1^) for 72 h at 22°C. The cells were harvested and downstream processing was carried out using cell death detection kit as per the manufacturer’s instructions (Roche). The cells (10,000 events per sample) were acquired on a BD FACS LSR II flow cytometer and histograms were generated using BD FACS DIVA software and MFI was recorded. The MFI values were further plotted to depict shift in MFI [24].

#### Cell cycle analysis

To ascertain DNA fragmentation, cell cycle analysis was performed that typically reveals cell population in G_0_/G_1_, S, and G_2_/M sub-phases of interphase stage, simultaneously also allowing the detection of apoptotic cells with fractional DNA content in pre- G_0_/G_1_ or sub- G_0_/G_1_ phase. The assay was done in accordance with Sarkar et al. [27] with few modifications. Parasites were cultured with or without CBD (34 μg ml^-1^) for 72 h, washed with PBS, fixed in 80% chilled ethanol and stored at 4°C for a minimum period of 24 h. Post-fixation, cells were subsequently washed, incubated with RNase (200 μg ml^-1^) for 1 h at 37°C, followed by staining with 50 μg ml^-1^ of PI for 20 min in dark. The cells were acquired using a BD FACS LSRII flow cytometer and the cell population in different interphase stages was depicted using BD FACS DIVA software.

### Evaluation of anti-amastigote efficacy of CBD and nitric oxide (NO) production from macrophages

The anti-amastigote potential of CBD was evaluated in *L*. *donovani* parasitized peritoneal macrophages isolated from BALB/c mice as described elsewhere [24]. The mouse peritoneal macrophages (5× 10^6^ cells ml^-1^) were seeded in 24–well plates (Corning, USA) and left for adherence for 24 h. After removal of non-adherent macrophages, the adherent cells were infected with *L*. *donovani* promastigotes (*Leishmania*: macrophage; 10:1) for a further 24 h in a CO_2_ incubator. Non-internalized promastigotes were removed by gentle washing and the infected macrophages were either left untreated or subjected to treatment with CBD or pentamidine (0–200 μg ml^-1^) at serial four fold dilutions. The drug treatment was carried out for 48 h following which the culture media was aspirated and the coverslips washed with PBS followed by fixation and giemsa staining. At least 200 macrophage nuclei per coverslip were counted and the number of resident amastigotes enumerated. Percent amastigote infectivity was determined using the formula:

$$\%\;\text{Infectivity}=\frac{\text{Number}\;\text{of}\;\text{amastigotes}\;\text{per}\;200{\text{macrophages}}_{\left(\text{treated}\;\text{samples}\right)}}{\text{Number}\;\text{of}\;\text{amastigotes}\;\text{per}\;200{\text{macrophages}}_{{}_{\left(\text{infected}\;\text{control}\right)}}}\times 100$$

IC_50_ was determined by graphical extrapolation.

To assay the effect of CBD on NO production, culture supernatants were collected after 48 h from CBD treated macrophages and from infected and uninfected controls, in parallel. The NO production was measured by assaying the nitrite (NO_2_^-^) content using Griess reaction as described elsewhere [28].

### Assessment of cytotoxicity against murine peritoneal macrophages *ex vivo*

The cytotoxicity of CBD against murine peritoneal macrophages was determined by MTT [3-(4,5-dimethylthiazol-2-yl)-2,5-diphenyl tetrazolium bromide] assay as described previously [24]. In brief, peritoneal macrophages (2×10^6^ ml^-1^) were seeded in 96 well tissue culture plates and left for adherence for 24 h in CO_2_ incubator. The non-adherent cells were removed by gentle washing with serum free media and the adherent macrophages were cultured in the absence or presence of serial two fold dilutions of CBD (0 to 500 μg ml^-1^) in triplicates. Post 48h, MTT reagent (5 mg ml^-1^) was added and formazan crystals were solubilized in isopropanol: dimethylsulfoxide (1:1). The plate was read at 570 nm in an ELISA plate reader. Macrophages without treatment served as control and their absorbance was considered as 100%. Percent viability for different experimental groups was calculated according to the formula:

$$\%\;\text{Viability}=\frac{{\text{Mean}\;\text{specific}\;\text{absorbance}}_{\left(\text{treated}\;\text{samples}\right)}}{{\text{Mean}\;\text{specific}\;\text{absorbance}}_{{}_{\left(\text{control}\;\text{samples}\right)}}}\times 100$$

### Identification of chemical constituents present in CBD by gas-chromatography mass spectrometry (GC-MS) analysis

GC-MS analysis was carried out to identify the plant secondary metabolites present in *C*. *cassia* bioactive fraction. Shimadzu QP2010 instrument quipped with DB-5 column (30m, film 0.25 μm, ID 0.25 mm) was used to carry out GC-MS. The column was heated gradually from 60°C to 310°C at 5°C min^-1^ and the injector and detector temperatures were kept at 260°C. Rest of the conditions were as elaborated previously [24] and the identification of plant secondary metabolites was performed by correlating the recorded mass spectra with those present in WILEY8.LIB and NIST08.LIB library provided along with the software of the GC-MS system.

### Evaluation of *in vivo* antileishmanial efficacy of CBD in *L*. *donovani* infected BALB/c mice

#### Mice infection and determination of parasite burden

6–8 weeks old BALB/c mice were infected with *L*. *donovani* promastigotes (2.5×10^7^/animal) via tail vein and infection was assessed in three arbitrarily selected animals eight weeks-post infection. Parasite burden in the host liver and spleen was assessed by examining the number of amastigotes in giemsa stained impression smears as well as parasite transformation from splenocytes *in vitro*. After confirmation of infection, infected mice (n = 5) were randomly assorted into different groups namely INF; infection control (infected mice that only received PBS), VC; vehicle control (that received 0.25% DMSO in PBS, the highest concentration used to dissolve the test fractions), CBD50 (CBD at 50 mg/kg bw) and CBD100 (CBD at 100 mg/kg bw) in parallel with uninfected control animals (normal group). AmB was used as a positive control in all the *in vivo* experiments and was reconstituted in sterile milli Q water containing 5% dextrose as described previously [25]. Treatment was carried out for ten days daily, the test fractions were administered orally and AmB (5 mg/kg bw) was administered intravenously.

Post-treatment, the animals were rested for one week following which mice from all the experimental groups were bled from retro-orbital plexus and euthanized for assessment of therapeutic and immunomodulatory efficacy of CBD. To estimate parasite burden, number of amastigotes per 500 macrophage nuclei were counted in giemsa stained dabbed-imprints of liver and spleen tissues. Parasite load was expressed as Leishman Donovan Units (LDU), which was calculated according to Bradley and Kirkley [29]:

$$\text{LDU}=\frac{\text{No}.\;\text{of}\;\text{amastigotes}\;}{\text{No}.\;\text{of}\;\text{macrophages}}\times \text{organ}\;\text{weight}(\text{mg})$$

Further, percent protection was deduced from respective LDU values according to the formula:

$$\frac{{\text{LDU}}_{\text{infected}\;\text{control}}-{\text{LDU}}_{\text{treated}}}{{\text{LDU}}_{\text{infected}\;\text{control}}}\times 100$$

### Analysis of T helper type (Th) 1 stimulatory potential of CBD in BALB/c mice

#### Preparation of leishmanial antigens

Leishmanial antigens were prepared as described earlier [25]. In brief, freeze thawed (FT) antigen was prepared from stationary phase promastigotes (10^8^ ml^-1^) which were harvested, washed with PBS and subjected to alternate freezing (-70°C, 30 min) and thawing (37°C, 15 min) for six cycles. The antigen thus prepared was quantitated by Folin and Lowry method [30]. Soluble leishmanial antigen (SLA) was also prepared in a similar manner; the stationary phase promastigotes (10^8^ ml^-1^) after eight cycles of freezing and thawing were centrifuged at 5250×g for 30 min and the supernatant collected. The antigen in the supernatant was quantified before storing it at -70°C until use.

#### Measurement of delayed type of hypersensitivity (DTH) response

Post one week of infection, all the experimental animals were inoculated with FT (40 μg/ 50 μl) in right hind footpad and an equivalent volume of PBS was injected in left hind footpad. The DTH response was analyzed in terms of difference in footpad swelling (right minus left) post 24 h of antigen administration [25].

#### Assessment of lymphoproliferation

Lymphoproliferative responses to SLA recall were studied in splenic lymphocytes. Spleens from all the experimental animals were removed, minced and single cell suspension prepared in serum free RPMI-1640 medium as described elsewhere [31]. The cells were resuspended at 5×10^6^ cells ml^-1^ in complete RPMI-1640 and cultured (37°C, 5% CO_2_) in the absence or presence of SLA (12 μg ml^-1^). Normal cells were also incubated in the presence of Concanavalin A (Con A, 5 μg ml^-1^), a non-specific mitogen that acted as positive control. Post 48 h, 5 mg ml^-1^ of MTT was added to cultures, the cells were further incubated for 4–6 h (37°C, 5% CO_2_), the plates were centrifuged (350 × g, 15 min) and the supernatant was carefully aspirated. The formazan crystals at the bottom of wells were dissolved in isopropanol:dimethylsulfoxide (1:1), the plate was read at 570nm and OD was recorded.

#### Estimation of T-helper type (Th)-1/Th2 cytokines

Post 48 h of SLA stimulation, the culture supernatants from all the experimental groups were collected and stored at -70°C until further use. The samples were retrieved and levels of interferon-gamma (IFN-γ), and interleukin (IL)-12p70 (Th1), IL-4 and IL-10 (Th2) cytokines were quantified using BD cytometry bead array kit as per the manufacturer’s instructions [31].

#### Determination of NO production

NO was estimated in culture supernatants of SLA stimulated or unstimulated splenocytes after 48 h of culture using Griess reagent as described elsewhere [24]. Sodium nitrite was used to generate a standard curve and nitrite concentration was calculated using specific OD values at 540 nm, generated in triplicates.

#### Analysis of serum Immunoglobulin G (IgG) isotypes

Levels of IgG2a and IgG1 were detected in sera from uninfected, untreated and treated mice by performing enzyme-linked immunosorbent assay (ELISA) as described elsewhere [25]. In brief, 96 well U-bottom plates coated with FT antigen (25 μg ml^-1^) were blocked with 1% BSA in PBS (blocking buffer) for 3–4 h at room temperature. The plates were then washed with washing buffer (PBS + 0.05% Tween-20) before the addition of primary antibodies (mice sera) at 1:1000 fold dilution. The plates were incubated overnight, washed followed by incubation with secondary antibodies (goat anti-mouse Ig2a and IgG1, Sigma). The plates were again washed and tertiary antibody (horseradish peroxidase conjugated rabbit anti-goat IgG) was added at 1:10,000 dilution. The substrate solution containing o-phenylenediamine dihydrocholride was prepared fresh and added to the plates. After ten minutes of incubation, absorbance was recorded at 450 nm in an ELISA plate reader.

### Determination of antileishmanial activity of CBD in hamster model of VL

Briefly, 4–6 weeks old male hamsters were infected intra-cardially by *L*. *donovani* promastigotes (2.5× 10^7^/animal). To ascertain the infection, post six weeks of infection, three hamsters were arbitrarily selected, euthanized and impression smears of liver and spleen prepared. Microscopic investigation of giemsa stained slides as well as transformation of splenic amastigotes into promastigotes under *in vitro* culture conditions confirmed the infection. The animals (n = 5) were then randomly assorted into different groups as mentioned for mice studies. Hamster dosing was performed daily for ten days according to dose and route of administration. After, one week of treatment, animals were bled by intra-cardiac puncture, sacrificed and parasite burden was determined as described previously [32].

### Assessment of correlates of cell-mediated immunity (CMI)

#### Estimation of DTH

DTH as an indicator of elicitation of CMI was evaluated in *L*. *donovani* infected hamsters one week post-treatment. Hamsters from all the experimental groups were injected with 50μg/50μl of FT in PBS intradermally in right food pad and PBS alone in the left footpad. The response was evaluated after 24 h by measuring the difference in footpad swelling (right minus left) with the aid of Vernier calipers as described elsewhere [32].

#### Analysis of lymphoproliferative responses

CFSE (carboxyfluoresceinsuccinimidyl ester) labeling was performed to assess *in-vitro* recall responses in SLA stimulated splenocytes. Splenocytes were resuspended at a cell density of 5×10^6^ cells ml^-1^ and labeled with CFSE (1μM) for 20 min in dark at room temperature. After incubation, complete RPMI-medium was added to quench the reaction and the cells were washed twice before seeding in the absence or presence of SLA (12 μg ml^-1^) or Con A (5 μg ml^-1^) [25]. Post 48 h, the cells were harvested and washed with PBS before acquisition in a BD LSR II flow cytometer. 50, 000 events were acquired for each sample and histogram analysis was performed with the help of BD FACS DIVA software.

#### Estimation of NO production

To elucidate the effect of bioactive fraction on NO production, culture supernatants were isolated from splenocytes incubated in the presence or absence of SLA (12 μg ml^-1^) for 48h in CO_2_ incubator. NO levels were then measured in terms of nitrite accumulation by Griess reagent as described elsewhere [24].

### Evaluation of hepatic and renal toxicity of CBD in experimental animals

Adverse effects of CBD treatment on host liver and kidney functions was evaluated in normal as well as *L*. *donovani* infected mice (n = 5). The mice were administered CBD at 100 mg/kg bw, or AmB (5 mg/kg bw) or DMSO (0.25% in PBS) as described above, daily for ten days. Post one-week, mice were bled and the sera analysed for the presence of serum glutamate pyruvate transaminase (SGPT), serum oxaloacetate transaminase (SGOT) and alkaline phosphatase (ALP). The serum enzyme concentrations were estimated as a marker for liver toxicity according to commercially available kits (Span Diagnostics Ltd., Surat, India). To assess any adverse effects on renal function, serum concentrations of creatinine and urea were estimated as per the manufacturer’s instructions (Span Diagnostics Ltd., Surat, India).

Toxicity profile of CBD was assessed in normal as well as infected hamsters (n = 3 to 5). The hamsters were fed CBD 100 mg/kg bw orally and the other control groups were treated as mentioned above. After one week, levels of SGOT, SGPT, and ALP along with urea and creatinine were estimated in the sera using commercially available kits (Span Diagnostics Ltd., Surat, India) as mentioned above.

### Statistical analysis

All the *in vitro* experiments were carried out thrice in triplicates. The results shown are from one of the three independent experiments performed and are expressed as mean ± standard error of mean of the samples in triplicate. The *in vivo* study was performed twice; five mice and hamsters per group were used in respective studies unless indicated. The data shown are from one of the two independent experiments and expressed as mean ± SEM. Graph-Pad Prism 5 software was employed for doing statistical analysis. Differences were considered statistically significant at *P*<0.05 and statistical significance was indicated with the help of appropriate symbols in graphs or explained in respective result section.

## Results

### Antileishmanial potential of *C*. *cassia* fractions

**Fig 1:**
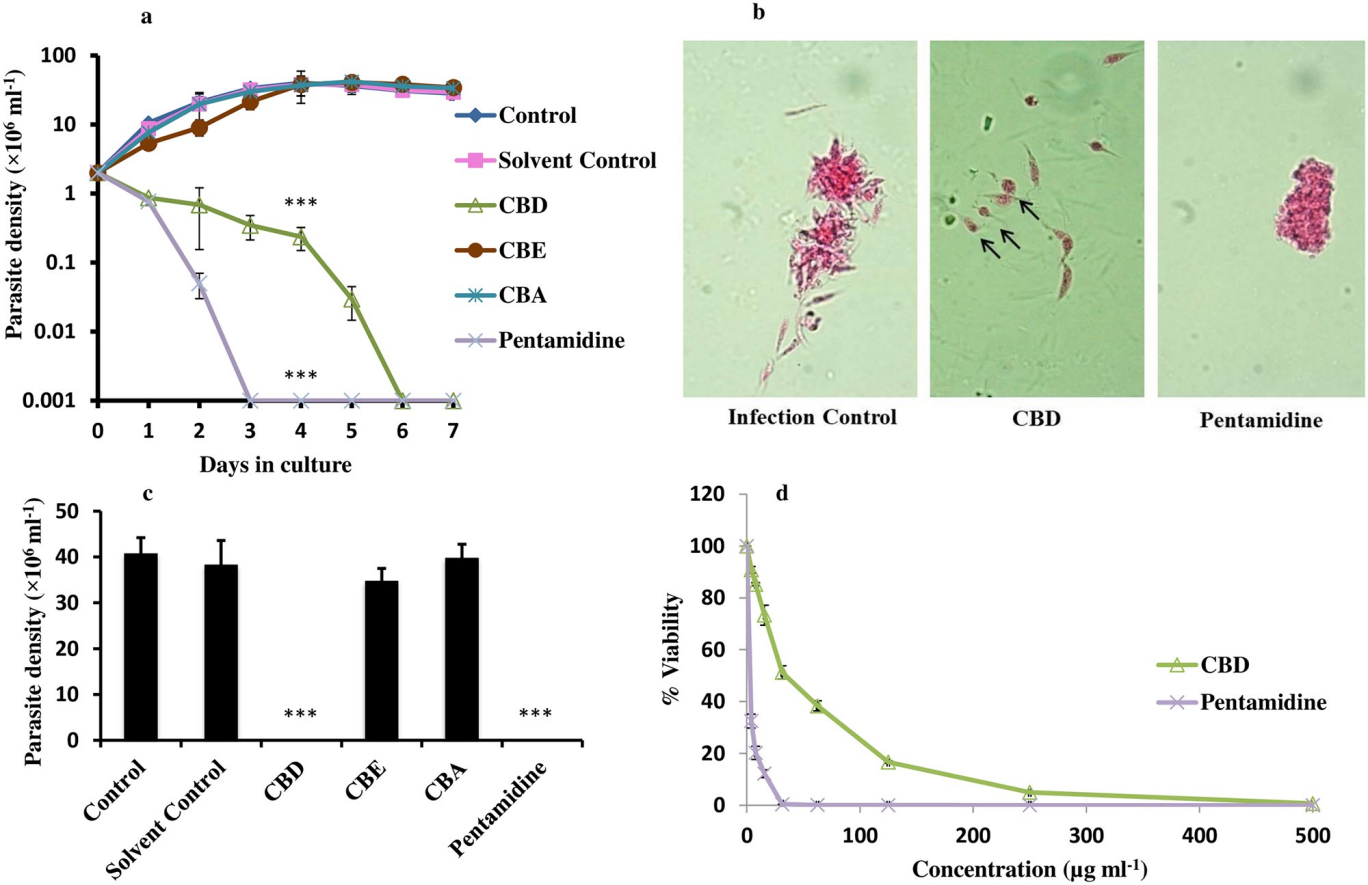
Anti-promastigote efficacy of *C*. *cassia* fractions. (a) Growth kinetics assay. Growth inhibitory potential of *C*. *cassia* fractions (500 μg ml^-1^) was assessed against *L*. *donovani* promastigotes (2×10^6^ ml^-1^). The parasite density was evaluated after every 24h as described in methods. Control = parasite control without any treatment; solvent control = 0.25% DMSO; CBD = *C*. *cassia* bark DCM fraction; CBE = *C*. *cassia* bark ethanolic fraction; CBA = *C*. *cassia* bark aqueous fraction. ***P<0.001 with respect to parasite control. (b) Morphological analysis of CBD treated promastigotes. Untreated or treated *L*. *donovani* parasites were stained with Erythrosin B and visualized under a phase contrast microscope at 100X. (c) Growth reversibility assay. *L*. *donovani* promastigotes (2× 10^6^ ml^-1^) were incubated with or without test fractions for 7 days and growth reversal after drug withdrawal was ascertained post 96 h. ***P<0.001with respect to parasite control. (d) IC_50_ determination. *L*. *donovani* promastigotes (2× 10^6^ ml^-1^) were incubated with CBD or pentamidine (500 μg ml^-1^) or medium alone at 22°C for 96 h after which the cell viability was determined as described in methods.

Anti-promastigote potential of *C*. *cassia* fractions were assessed against *L*. *donovani* promastigotes. CBD profoundly declined the growth of *L*. *donovani* promastigotes (*P*<0.001) *in vitro* (Fig 1a). In case of ethanolic (CBE) and aqueous (CBA) fractions, no significant suppression in parasite growth (*P*>0.05) was visible. Solvent control (0.25% DMSO) was also inert in arresting the parasite growth while pentamidine completely inhibited the growth of parasites at three days of culture (*P*<0.001). CBD treated parasites lost their typical shape and became reduced in size with absent or shortened flagella post 96h of CBD treatment. In contrast, untreated parasites retained their flagellated and elongated shape. Pentamidine treated parasites were clumped with each other, reduced in size, and had no flagella (Fig 1b).

To assess whether the mode of induced cell death by the bioactive fractions was cytostatic or cytocidal, the treated and untreated *L*. *donovani* promastigotes were washed to remove the drug and re-incubated in fresh M199 media for 96 h. Analysis of cell count post-drug withdrawal, revealed that there was no reversion of growth in CBD treated sample (*P*<0.001) indicating its leishmanicidal effect. Substantial reversion in parasite growth was evident in all the other (Fig 1c) groups (*P*>0.05) except in pentamidine treated samples wherein no reversal in parasite growth was observed (*P*<0.001).

IC_50_ of CBD was determined by incubating the parasites with serial two-fold dilution of respective fractions or compounds. CBD induced a dose dependent reduction in parasite viability with IC_50_ of 33.66±3.25 μg ml^-1^. IC_50_ achieved with pentamidine was 1.09±0.055 (Fig 1d).

### Induction of apoptosis in CBD-treated *L*. *donovani* promastigotes

#### Phosphatidylserine externalization post treatment with CBD

**Fig 2:**
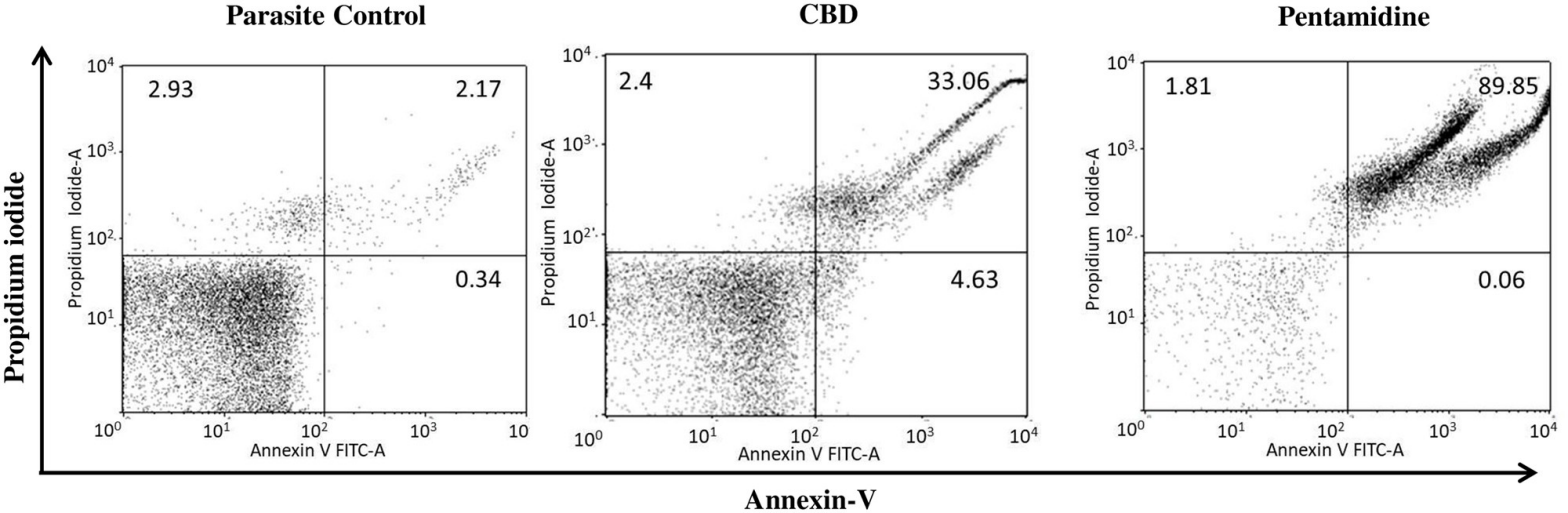
Induction of PS externalization in *L*. *donovani* promastigotes. CBD or pentamidine (34 μg ml^-1^) treated promastigotes (2×10^6^ ml^-1^) were co-stained with Annexin-V-FLUOS and PI. The cells were acquired on a flow cytometer and dot plots generated. The lower left quadrant represents the percentage of live cells (Annexin-V and PI dual negative), whereas apoptotic cells (Annexin-V positive) can be seen in lower right quadrant. The upper left quadrant represents apoptotic (or PI positive) cells whereas upper right quadrant represents late apoptotic cells (Annexin-V and PI dual positive). The percentage of stained cells is mentioned in respective quadrants.

PS externalization was visualized by co-staining with Annexin-PI post CBD treatment. In CBD treated cells, 33.06% cells were found to be secondary apoptotic and 4.63% cells were found to be Annexin-V positive. A small percentage of cells (2.4%) were PI positive which indicates compromised membrane integrity by CBD treatment. A marginal fraction of cells (5.44%) underwent cell death in untreated control. Together, the data indicates that CBD induces apoptosis in *L*. *donovani* promastigotes (Fig 2).

#### Changes in Ψm post-treatment with CBD

**Fig 3:**
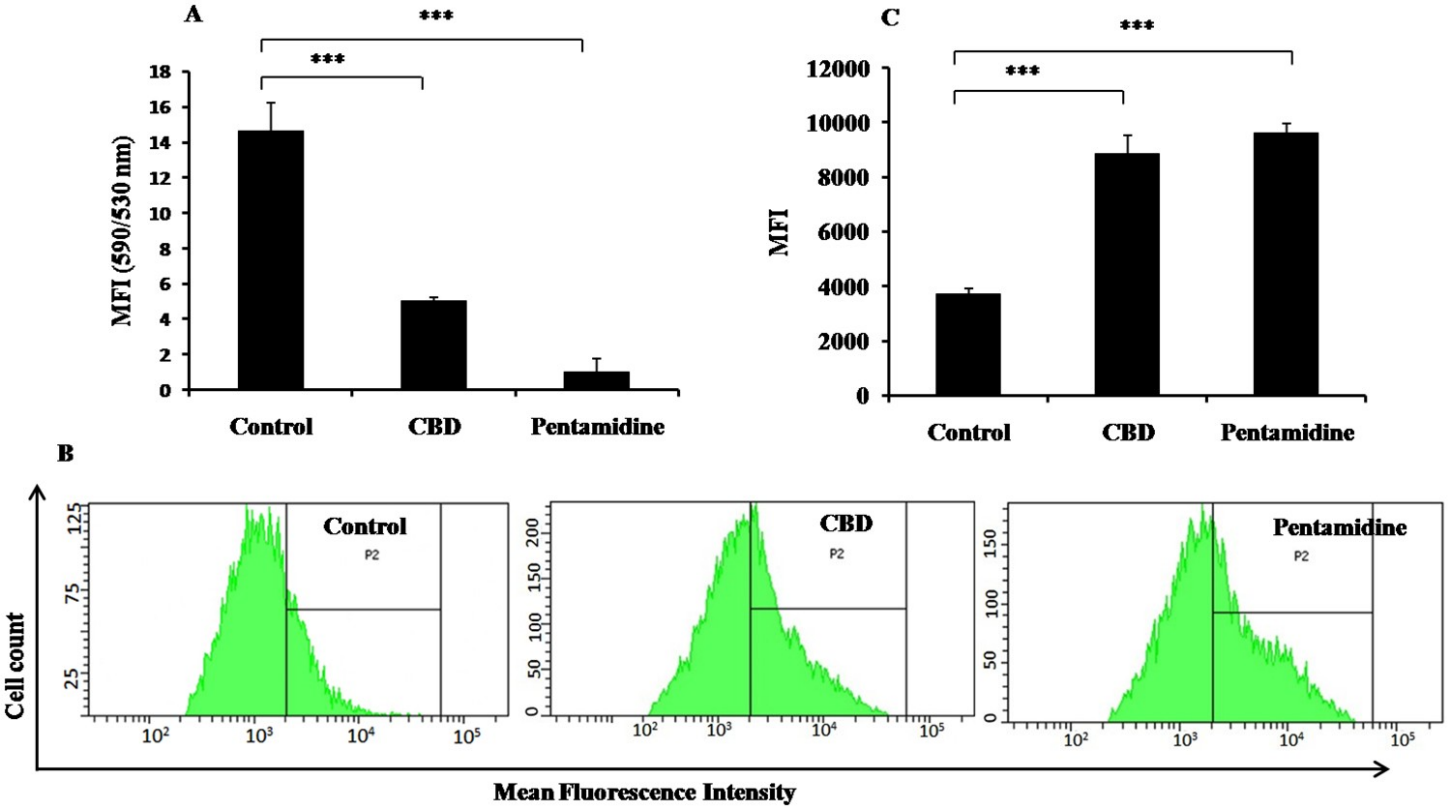
Apoptotic events in *L*. *donovani* promastigotes. (a) Study of mitochondrial membrane potential. *L*. *donovani* promastigotes (2×10^6^ ml^-1^) were subjected to treatment with *C*. *cassia* bioactive fraction and pentamidine (34 μg ml^-1^) for 72 h at 22°C. The untreated and treated parasites were stained with JC-1 as described in methods. ***P<0.001 with respect to parasite control. (b) Determination of ROS generation. Parasites (2× 10^6^ ml^-1^) were incubated with or without CBD and pentamidine (34 μg ml^-1^, 72 h, 22°C) and stained with H_2_DCFDA for detection of ROS production. Shift in mean fluorescence intensity (MFI) in P2 gated region depicted the extent of ROS production. (c) Bar graph representing changes in ROS production. The recorded MFI in untreated or treated samples are represented graphically to divulge the changes in ROS production. ***P<0.001 with respect to parasite control.

Loss of mitochondrial membrane potential is critical for initiation of apoptosis. The fall in Ψm is represented by decline in J-aggregates/J-monomers or red (590)/ green (530) ratio as JC-1 fluoresces red in live and green in cells undergoing apoptosis. There was a significant fall in relative Ψm of *Leishmania* parasites after treatment with CBD (5.02±0.20, *P*<0.001) and pentamidine (1.00±0.76, *P*<0.001) in comparison to control (14.62±1.58) indicating mitochondrial membrane depolarization in treated samples (Fig 3a).

#### Generation of ROS post-treatment with CBD

Commencement of apoptosis is also dependent upon ROS generation, which helps in both initiation and execution of apoptosis. To calculate the shift in MFI, P2 gate was positioned according to the placement of unstained control population (negative control) and stained positive control population. ROS generation was found to be considerably enhanced in CBD treated promastigotes (MFI = 8860, *P*<0.001) with respect to control cells (Fig 3b and 3c) without any treatment (MFI = 3739). Pentamidine treatment also enhanced ROS generation significantly (MFI = 9603, *P*<0.001).

#### DNA fragmentation in CBD treated promastigotes

**Fig 4:**
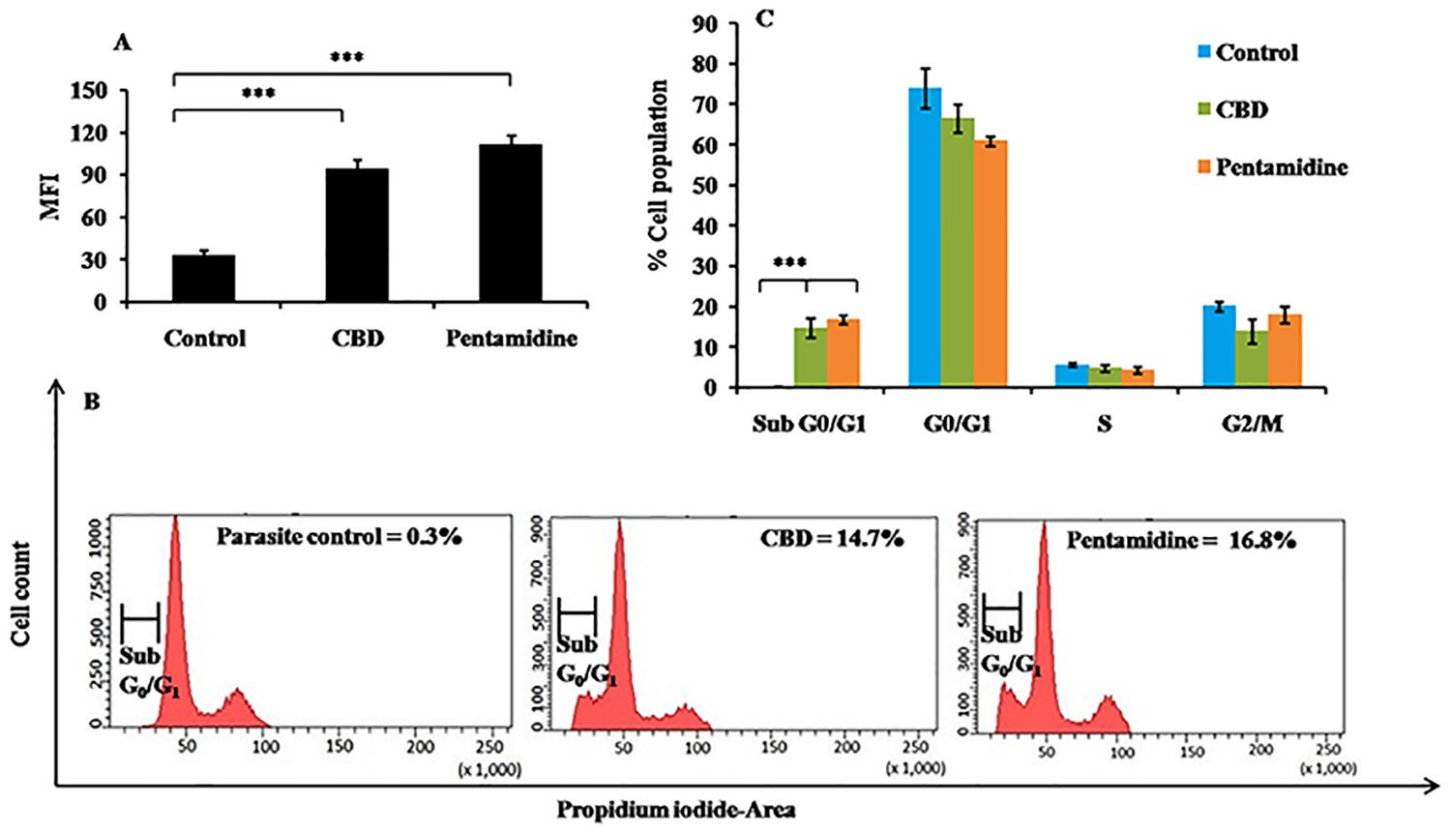
DNA fragmentation and cell cycle analysis. (a) Detection of DNA fragmentation by TUNEL assay. Parasites (2×10^6^ ml^-1^) were cultured with CBD and pentamidine (34 μg ml^-1^) for 72 h at 22°C. The TUNEL staining was carried out as described in methods. ***P<0.001 with respect to parasite control. (b) Cell cycle analysis. *L*. *donovani* promastigotes (2×10^6^ ml^-1^) were treated with test fraction and pentamidine (34 μg ml^-1^). Post 72 h, the cells were processed for PI staining and flow cytometry analysis as described in methods. (c) Bar graph depicting changes in cell population in different stages of interphase in treated as well as untreated samples. ***P<0.001 with respect to parasite control.

The DNA fragmentation which is also one of the hallmark features of apoptosis was documented through TUNEL labeling, wherein nicked ends of fragmented DNA were detected by addition of FLOUS labelled dUTPs by Tdt enzyme. The histogram analysis revealed that only baseline MFI = 33.05, was observed in control samples whereas in pentamidine treated samples, the MFI recorded was 111.73 (*P*<0.001). In CBD treated samples, the MFI increased to 94.22 (*P*<0.001) with respect to control, demonstrating apoptosis inducing capacity of CBD (Fig 4a).

#### Influence of CBD treatment on sub G_0_/G_1_ population

An increase in DNA content in sub G_0_/G_1_ phase is indicative of DNA fragmentation, which in turn signifies occurrence of apoptosis. In control group, only 0.3% cells were detected in sub G_0_/G_1_ phase (Fig 4b and 4c) and the cell population was notably increased to 14.7% (*P*<0.001) after CBD treatment. Pentamidine treatment also enhanced the percentage of apoptotic cells up to 16.8% (*P*<0.001).

### Anti-amastigote potential of CBD, NO production and cytotoxicity *ex vivo*

**Fig 5:**
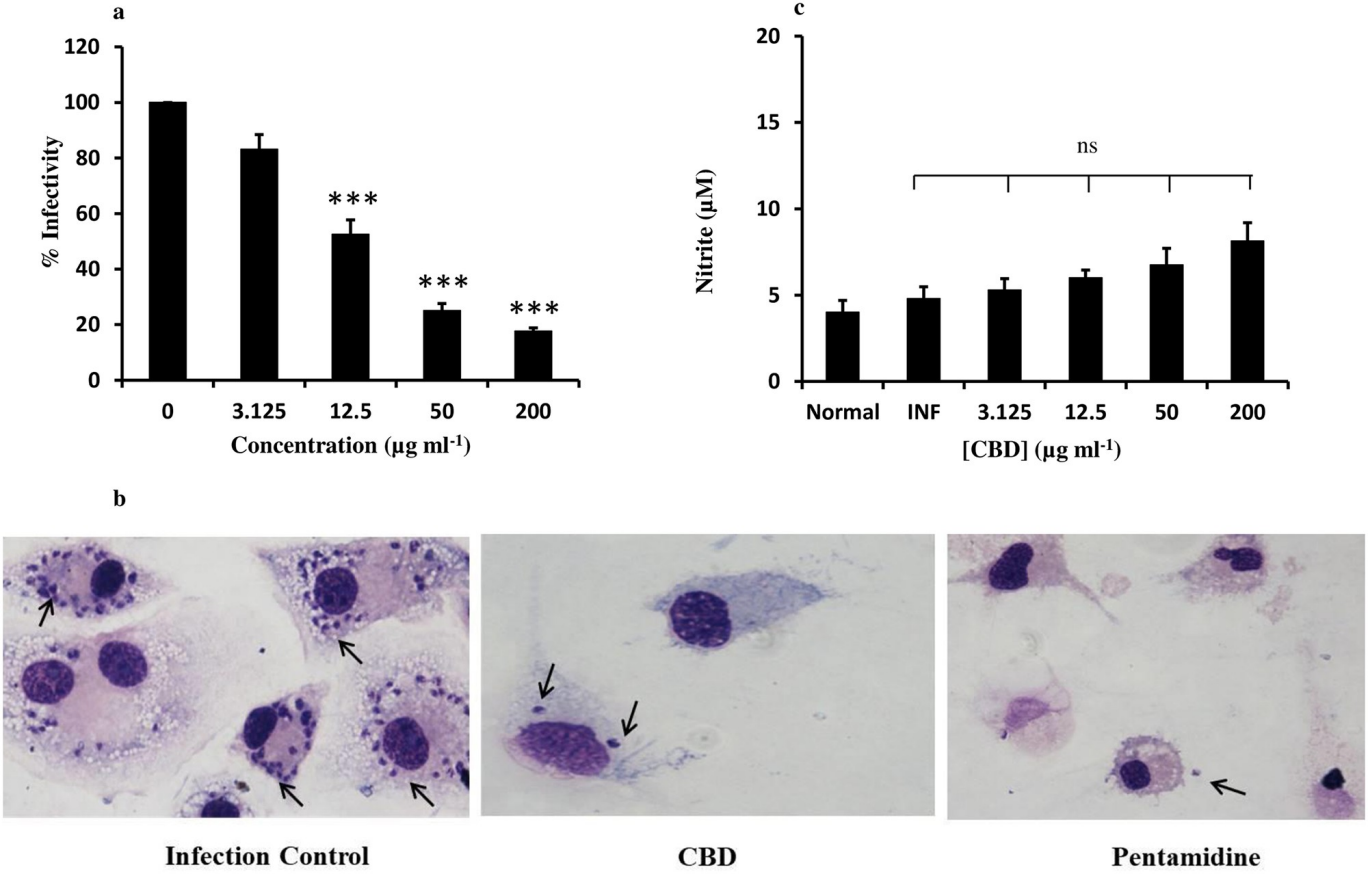
Effect of CBD treatment on *L*. *donovani* infected/ normal peritoneal macrophages. (a) Anti-amastigote potential of CBD. Peritoneal macrophages (5×10^6^ ml^-1^) were infected with *Leishmania* parasites (10:1). Promastigotes were allowed to transform into amastigotes under optimum *ex vivo* conditions (37°C, 5% CO_2_) and were treated with CBD (0–200 μg ml^-1^). Post 48 h, the amastigote number and percent infectivity was calculated as described in methods. ***P<0.001 with respect to parasite control. (b) Detection of NO generation. *L*. *donovani* infected macrophages were treated with CBD and culture supernatants were collected after 48 h. NO production was quantified in terms of nitrite by means of Griess reagent as depicted in methods. ns = *P*>0.05 with respect to infection control group. (c) Microscopic imaging of Giemsa stained *Leishmania* infected peritoneal macrophages. The infected macrophages were subjected to treatment with CBD or pentamidine for 48 h after which the cells washed, fixed and giemsa stained as described in methods. The infection control group bore high amastigote burden whereas CBD or pentamidine treatment profoundly reduced the amastigote burden (as indicated by arrows).

The anti-amastigote efficacy of *C*. *cassia* bioactive fraction was evaluated in *L*. *donovani* infected peritoneal macrophages derived from BALB/c mice. CBD was found to be effectual in reducing *L*. *donovani* amastigote infection *ex vivo* with IC_50_ of 14.06±2.46 μg ml^-1^ while that of pentamidine was observed to be 0.85±0.02 μg ml^-1^ (Fig 5a). The reduction in parasite burden post CBD treatment was also evident in giemsa stained photomicrographs taken at 100X under oil immersion in a phase contrast microscope (Fig 5b).

Effect of CBD treatment on NO production was assessed in culture supernatants of uninfected, infected and variously treated macrophages. CBD treatment (200 μg ml^-1^) enhanced the NO production in an inconspicuous manner by ~1.69 folds (*P*>0.05) in comparison to infected controls (4.79 μM). Basal level of NO production was observed in normal uninfected macrophages (4 μM) while none of the lower concentrations of CBD significantly impacted the NO production (Fig 5c).

**Fig 6:**
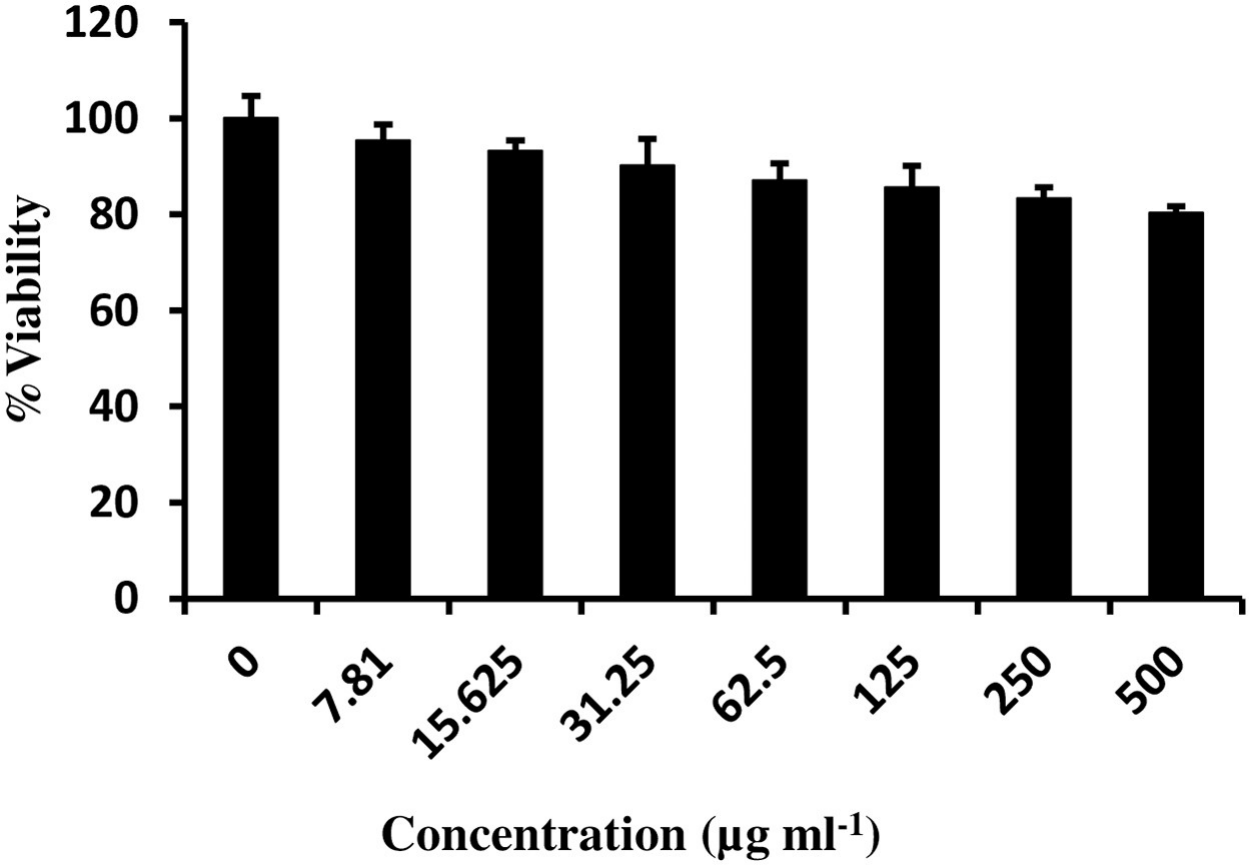
Cytotoxicity of CBD against peritoneal macrophages. BALB/c mice peritoneal macrophages (2×10^6^ ml^-1^) were isolated and seeded in 96 well plates for determination of cell viability post CBD treatment (0–500 μg ml^-1^, 48h) by MTT assay. The plate was read at 570 nm and absorbance was recorded to determine percent viability as described in methods.

*In vitro* cytotoxicity of *C*. *cassia* bioactive fraction was evaluated against peritoneal macrophages isolated from normal BALB/c mice. MTT assay was performed to assess the cell viability, and the data obtained indicated no apparent decline in cell viability at any of the concentrations tested (Fig 6). This indicated that *C*. *cassia* lacked any cytotoxicity against peritoneal macrophages even at the highest concentration of 500 μg ml^-1^.

### GC-MS analysis of CBD

GC-MS was performed to identify the chemical constituents present in CBD. A total of 118 compounds were detected which are listed in S1 Table. Cinnanmaldehyde (36.27%), cinnamaldehyde dimethyl acetal (21.52%), 1,2-Benzenedicarboxylic acid (10.92%) and o-methoxycinnamaldehyde (5.58%) were detected in major proportions along with other compounds listed in Table 1.

**Table 1:** Major plant secondary metabolites present in CBD.

S. No.	Retention Time	%Area	Compound
**1**.	14.163	36.27	Cinnamaldehyde
**2**.	17.239	21.52	Cinnamaldehyde dimethyl acetal
**3**.	20.439	5.58	o-Methoxycinnamaldehyde
**4**.	39.516	10.92	1,2-Benzenedicarboxylic acid
**5**.	18.162	2.81	Coumarin
**6**.	22.493	2.04	Viridiflorol

### *In vivo* therapeutic efficacy of CBD in *L*. *donovani* infected mice

**Fig 7:**
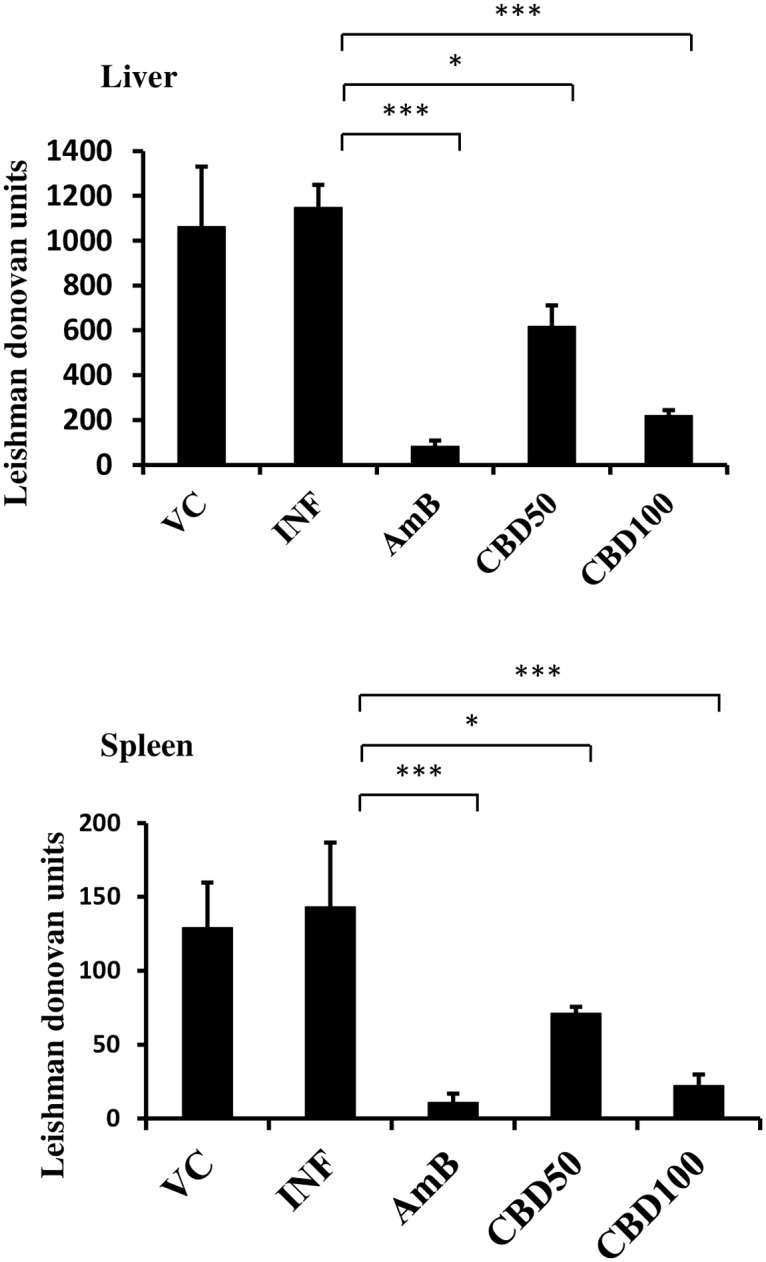
Therapeutic efficacy of CBD in *L*. *donovani* infected BALB/c mice. *L*. *donovani* infected BALB/c mice were either untreated (INF, infection control group) or treated with VC (vehicle control), AmB (Amphotericin B, 5 mg/kg bw), CBD50 (*C*. *cassia* bark DCM fraction at 50 mg/kg bw) and CBD100 (*C*. *cassia* bark DCM fraction at 100 mg/kg bw). The parasite burden in host liver and spleen was calculated and expressed as Leishman Donovan Units. *P<0.05 and ***P<0.001 with respect to infection control.

The *in vivo* antileishmanial effectualness of CBD was assessed at 50 and 100 mg/kg bw dose in *L*. *donovani* infected BALB/c mice. Heavy parasitic load was detected in host liver and spleen (Fig 7). Marginal levels of protection were witnessed in VC control group with percent reduction in parasite load being as low as 7.43% and 6.54% in liver and spleen, (*P*>0.05) respectively. CBD treatment at 100 mg/kg bw rendered significant protection corresponding to 80.91% (liver, *P*<0.001) and 82.92% (spleen, *P*<0.001). Treatment at 50 mg/kg bw CBD rendered partial (liver = 46.34%; spleen = 47.95%) but significant (*P*<0.05) protection whereas AmB treatment (liver = 92.88%, spleen = 93.09%) resulted in substantial protection (*P*<0.001) to *L*. *donovani* infected mice.

### Th1 stimulatory potential of CBD in BALB/c mice

#### DTH responses post-treatment with CBD

**Fig 8:**
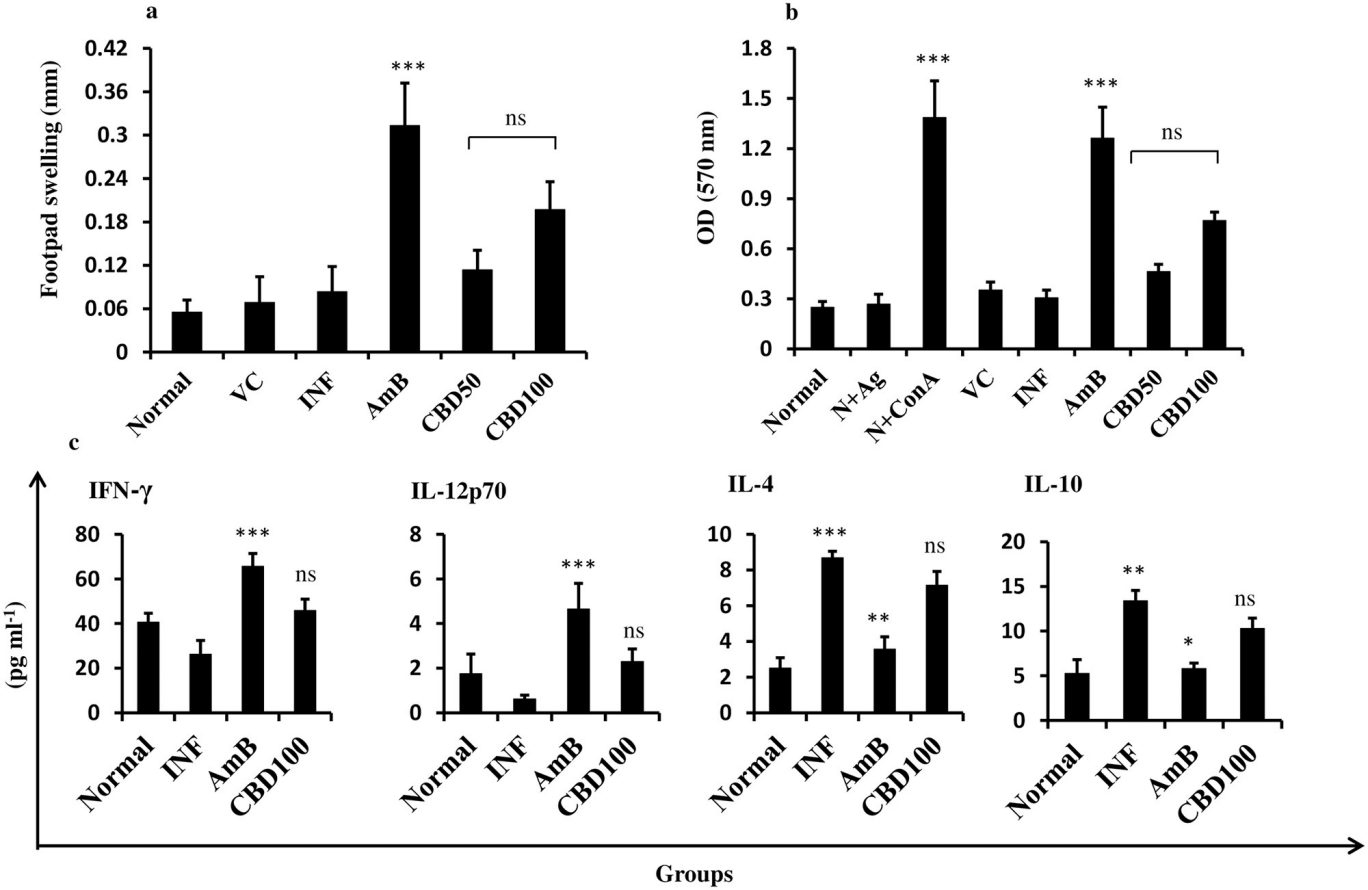
Evaluation of pro-inflammatory potential of CBD in *L*. *donovani* infected BALB/c mice. (a) DTH response post CBD treatment. Magnitude of DTH was evaluated post 24 h of FT antigen inoculation in uninfected (normal group), untreated and variously treated groups. ***P<0.001 with respect to infection control. (b) Assessment of lymphoproliferation. Lymphoproliferative responses generated in unstimulated or SLA stimulated splenocytes from different treatment groups was analyzed by MTT assay as described in methods. ***P<0.001 in comparison to infection control. (c) Analysis of Th1/Th2 cytokine production. Post CBD (100 mg/kg bw) treatment levels of Th1 and Th2 cytokines were evaluated in culture supernatants of splenocytes post 48h of SLA stimulation. *P<0.05, **P<0.001 and ***P<0.001 and ns = non-significant in comparison to infection control.

DTH response as a correlate of CMI was evaluated in normal, infected and variously treated mice (Fig 8a). CBD treatment at 100 and 50 mg/kg bw only partially stimulated DTH (*P*>0.05) whereas recovery from infection after AmB treatment was accompanied by generation of strong DTH response (*P*<0.001).

#### *In vitro* recall responses in BALB/c mice splenocytes

Splenocytes from all the experimental groups were isolated and *in vitro* restimulated with SLA to study lymphoproliferation. Basal levels of lymphoproliferation were observed in normal cells (Fig 8b), which was not significantly altered after treatment with CBD at the indicated doses (*P*>0.05). Con A (positive control) stimulated cells rapidly proliferated (*P*<0.001) and AmB (5 mg/kg bw) treated animals also exhibited strong lymphoproliferative responses (*P*<0.001).

#### Alterations in Th1/Th2 cytokine levels post CBD treatment

Changes in pro-inflammatory (Th1) and anti-inflammatory (Th2) cytokine levels were detected post-treatment with *C*. *cassia* bioactive fraction. INF-γ and IL-12p70 levels which were down modulated in INF group (Fig 8c) where restored to normal range after CBD treatment (*P*>0.05) at 100 mg/kg bw dose. AmB treatment substantially elevated INF-γ and IL-12p70 levels (*P*<0.001) in comparison to untreated animals (infection control group). IL-4 and IL-10 levels were found to be significantly enhanced in infected animals in comparison to normal mice (*P*<0.01). No noticeable alterations in IL-4 and IL-10 levels were evident after CBD 100 mg/kg bw treatment (*P*<0.05).

#### NO production in *L*. *donovani* infected BALB/c mice

**Fig 9:**
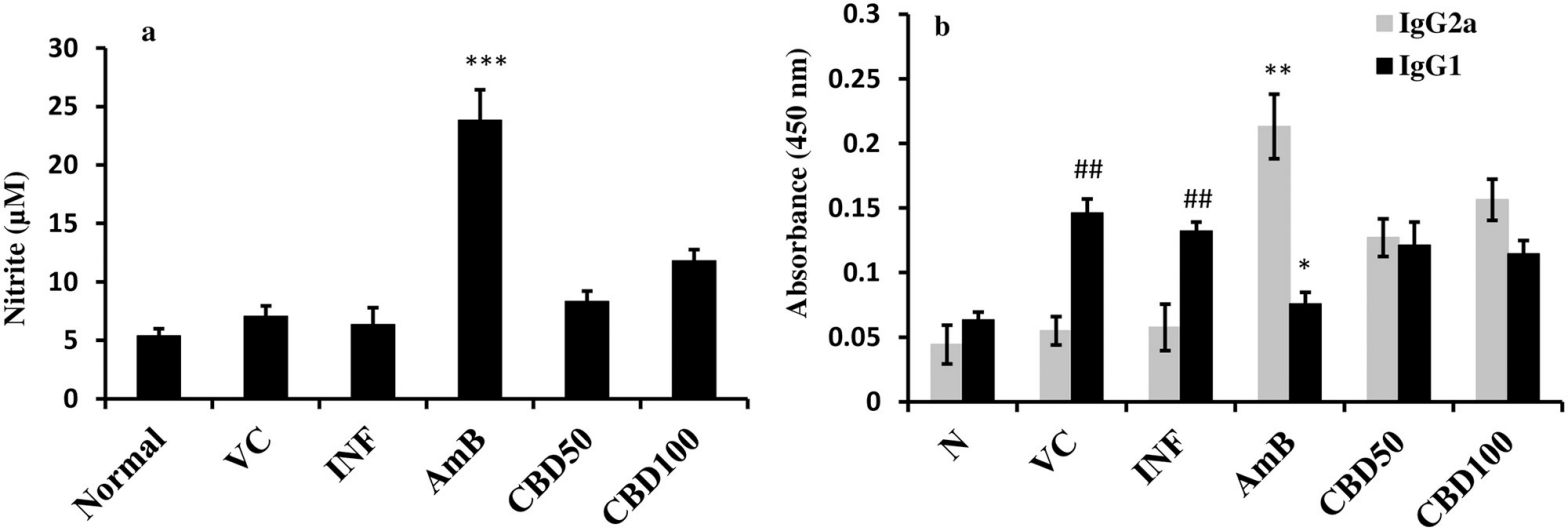
Analysis of NO production and IgG subclass response. (a) Estimation of NO production in BALB/c mice splenocytes. NO production was quantified in terms of nitrite in culture supernatants of SLA stimulated splenocytes as described in methods. ***P<0.001 with respect to infection control. (b) Determination of serum levels of IgG isotypes. IgG2a and IgG1 levels were determined in mice sera from different experimental groups by ELISA as explained in methodology. IgG1 was particularly enhanced in VC and INF group in comparison to normal mice (##*P*<0.01). *P<0.05 and**P<0.001 in comparison to infection control.

NO production was analyzed post 48 h in SLA stimulated splenocytes from all the experimental groups (Fig 9a). NO quantification in terms of nitrite levels revealed that CBD treatment at 50 and 100 mg/kg bw doses exhibited marginal effect (*P*>0.05) whereas NO production was conspicuously increased after AmB treatment (*P*<0.001).

#### Analysis of *Leishmania*-specific antibody responses

Serum levels of IgG isotypes *i*.*e*., IgG2a and IgG1 as surrogate markers for Th1/Th2 immune response were analysed. The antibody response was measured through ELISA (Fig 9b). IgG2a levels were partially stimulated post CBD (100 mg/kg bw) treatment though the enhancement was non-significant in comparison to infection control group (*P*>0.05). The change in IgG2a levels was less noticeable at 50 mg/kg bw dose (*P*>0.05). IgG1 levels were found to be significantly elevated in infected and vehicle control treated animals (*P*<0.01). No significant decline in IgG1 levels was evident after CBD (50 and 100 mg/kg bw) treatment unlike AmB which successfully declined IgG1 (*P*<0.05) and upregulated IgG2a levels (*P*<0.01).

### Therapeutic efficacy of *C*. *cassia* bioactive fraction in *L*. *donovani* infected hamsters

**Fig 10:**
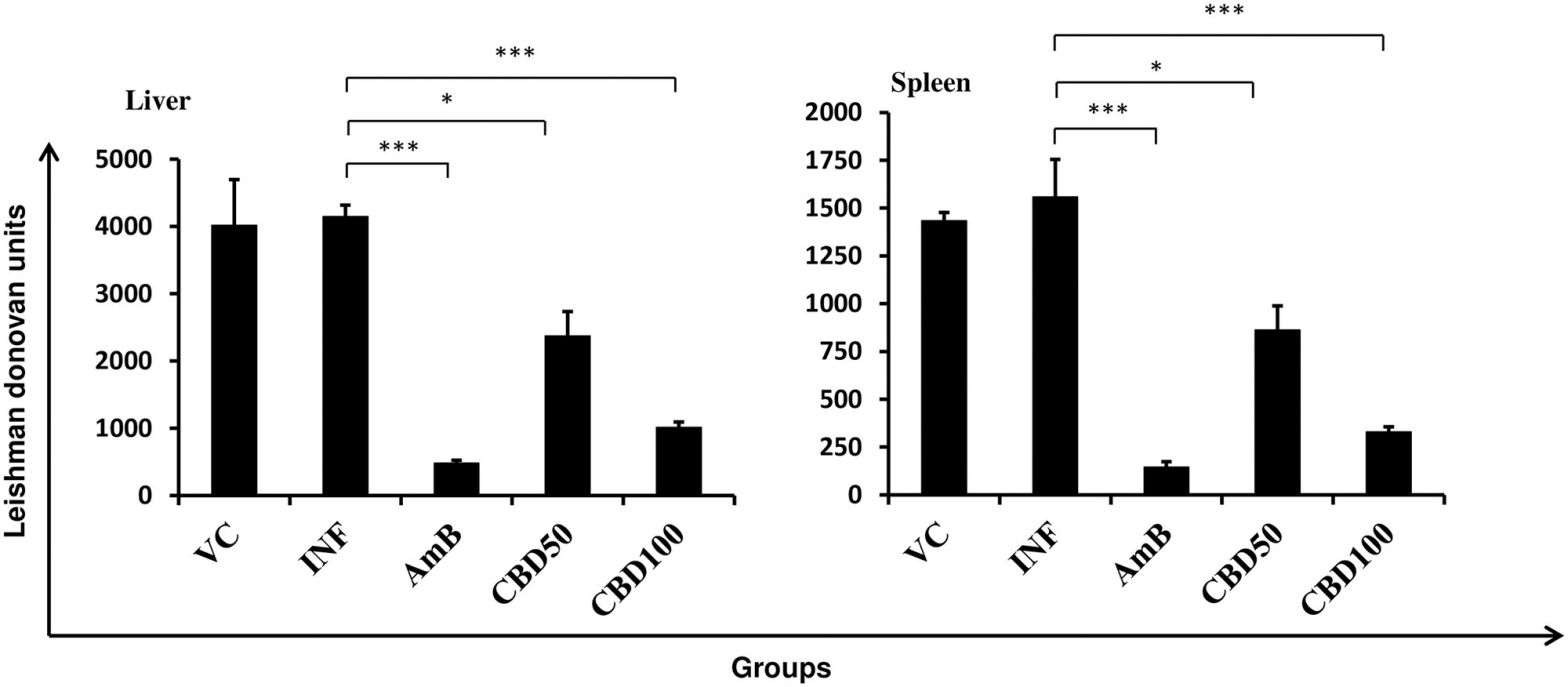
Antileishmanial efficacy of *C*. *cassia* bioactive fraction in *L*. *donovani* infected hamsters. 4–6 week old male hamsters were infected with *L*. *donovani* promastigotes and post six weeks of infection the hamsters were treated orally with CBD for ten days. One week post-treatment animals were sacrificed and parasite burden was estimated in giemsa stained impression smears of host liver and spleen. *P<0.05 and ***P<0.001 with respect to infection control.

*In vivo* antileishmanial efficacy of CBD was also examined in hamster model of the disease. 4–6 week old male hamsters infected with *L*. *donovani* parasites were treated with CBD at 50 and 100 mg/kg bw. High parasitic load was detected in untreated hamsters with LDU in liver and spleen corresponding to 4147.75±171.11 and 1433.69±197.59, respectively. Vehicle control group conferred least protection to *L*. *donovani* infected hamsters (Fig 10). CBD treatment at 100 mg/kg bw significantly declined the parasite burden (*P*<0.001) with 75.61% and 78.93% protection in liver and spleen, respectively. At 50 mg/kg bw, the reduction in parasite burden was partial but significant (*P*<0.05) in comparison to infection control group and 42.92% protection was achieved in case of liver whereas 44.64% was observed in spleen. AmB treatment substantially decreased the parasite burden (*P*<0.001) and conferred 88.51% (liver) and 90.71% (spleen) protection.

### Correlates of CMI in Hamsters

#### DTH response in *L*. *donovani* infected hamsters

**Fig 11:**
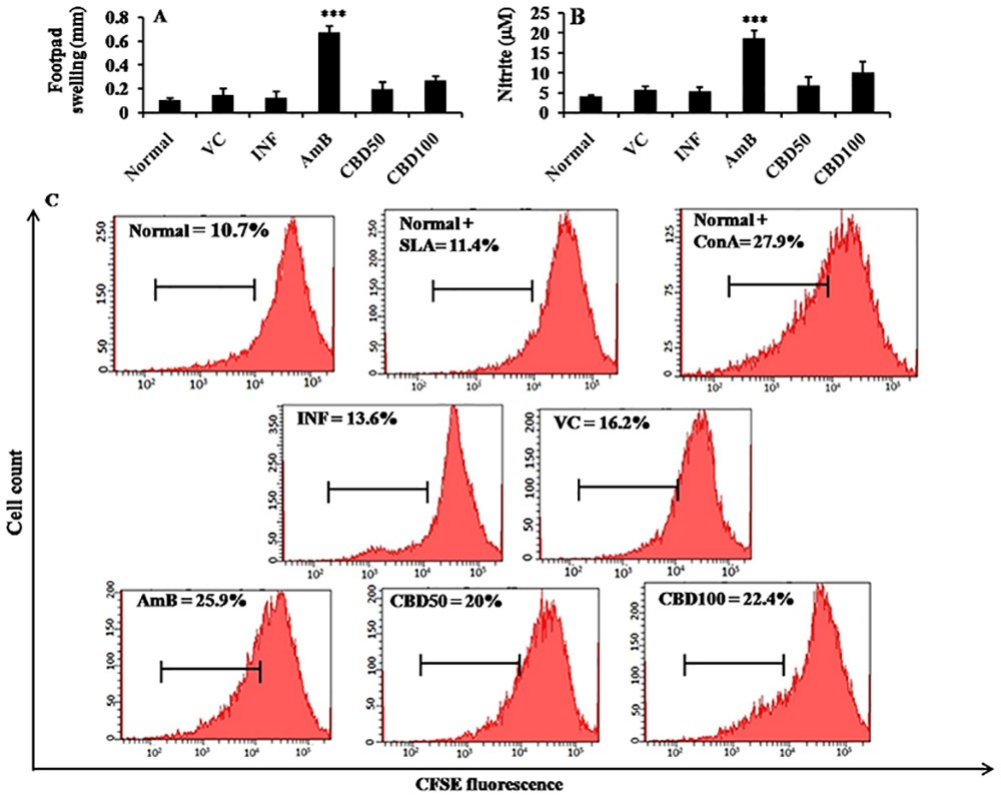
Evaluation of correlates of CMI in *L*. *donovani* infected hamsters. (a) DTH response in CBD treated hamsters. Hamsters were inoculated with FT and PBS in right left hind footpads respectively. Post 24 h, differences in the footpad swelling (right-left) were calculated and mean±SEM were plotted to analyze the generation of DTH responses. ***P<0.001 in comparison to infection control. (b) NO production in *L*. *donovani* infected hamsters. NO generation in different experimental groups was studied by means of Griess reaction as described in methods. ***P<0.001 with respect to infection control. (c) *In vitro* recall responses in SLA stimulated splenocytes. Hamster splenocytes were labeled with CFSE and cultured in the absence or presence of SLA for 48 h. CFSE fluorescence was analyzed on BD LSR II flow cytometer and BD FACS DIVA software was used to generate histograms where percentage CFSE positive cells (as indicated) was deduced from histogram statistics.

DTH response post-CBD treatment was studied by inoculating the hamster hind footpads with leishmanial FT antigen (Fig 11a). The footpad swelling was only partially enhanced (~2.18 folds, *P*>0.05) after CBD 100 mg/kg bw treatment. CBD treatment at 50 mg/kg bw induced non-significant DTH response reflected by low increase (~1.58 folds, *P*>0.05) in footpad thickness. AmB treatment generated strong DTH responses in hamsters (~5.51 folds, *P*<0.001) in comparison to infected mice.

#### NO generation post-CBD treatment

Changes in NO production were analyzed by Griess assay in culture supernatant of SLA stimulated splenocytes. The augmentation in NO generation was non-significant (~1.87 folds, *P*>0.05) after CBD 100 mg/kg bw treatment in comparison to infection control. At 50 mg/kg bw treatment, the change in NO level was negligible (Fig 11b) whereas AmB treatment siginificantly elevated NO generation (~3.46 folds, *P*<0.001).

#### Lymphoproliferative responses in hamsters

Lymphoproliferative responses were studied by labeling the splenocytes with CFSE before culturing them in the presence or absence of SLA. CFSE fluorescence becomes half of its original value after every cell division and thus a shift or decrease in fluorescence towards left hand side of the axis indicates cell proliferation. Basal levels of lymphoproliferation were observed in normal group with or without antigen stimulation (Fig 11c). 13.6±2.60% and 16.2±1.70% cells were found to be CFSE positive in infection and vehicle control group, respectively. Cells was partially stimulated in CBD (100 mg/kg bw) treated mice (22.4±1.77%, *P*>0.05) and at 50 mg/kg bw dose, percentage of proliferating cells was found to be 20±1.57% (*P*>0.05). Con A stimulation (27.9±1.76%, *P*<0.001) and AmB treatment (25.9±1.69, *P*<0.001) significantly enhanced the lymphoproliferation.

#### *In vivo* side effects of CBD treatment on hepatic and renal functions

*In vivo* cytotoxicity of CBD was evaluated both in normal and infected BALB/c mice at 100 mg/kg bw dose. CBD treatment did not significantly alter serum SGOT, SGPT and ALP levels along with creatinine and urea in normal mice, indicating its inertness (Table 2). AmB treatment also did not induce any substantial changes in serum levels of liver enzymes and renal metabolites in comparison to normal mice (*P*>0.05). However, in case of *L*. *donovani* infected mice, the serum levels of SGOT and SGPT were found to be particularly enhanced in infection and vehicle control group (*P*<0.001) which were brought down to normal range after CBD treatment (Table 3). Neither ALP nor creatinine and urea levels varied significantly from normal range in any of the experimental groups. AmB treatment also did not adversely impact host hepatic and renal functions.

**Table 2:** *In vivo* toxicity of CBD in BALB/c mice.

Effect on liver and kidney functions of normal BALB/c mice					
Groups	SGOT (U/L)	SGPT (U/L)	ALP (KA)	Creatinine (mg/dl)	Urea (mg/dl)
**Normal**	24.45±5.19	30.59±6.93	5.36±1.32	1.32±0.22	35.12±4.10
**AmB**	28.92±3.21	36.32±4.9	4.65±0.72	1.11±0.12	30.12±3.38
**CBD100**	27.65±4.32	35.5±4.73	5.31±0.29	1.35±0.25	31.14±5.36

**Table 3:** *In vivo* toxicity of CBD in *L*. *donovani* infected mice.

Effect on liver and kidney functions of infected BALB/c mice					
Groups	SGOT (U/L)	SGPT (U/L)	ALP (KA)	Creatinine (mg/dl)	Urea (mg/dl)
**Normal**	27.54±6.21	45.54±4.23	5.06±0.43	3.33±0.12	25.55±8.87
**INF**	79.91±3.65^+++^	98.68±8.21^+++^	3.06±0.98	4.55±0.36	38.57±5.66
**VC**	71.40±6.31^+++^	109.89±10.6^+++^	3.68±0.89	4.12±0.57	41.11±9.25
**AmB**	38.12±2.89***	62.23±8.89**	6.74±0.50	2.22±0.36	32.22±4.96
**CBD100**	34.96±4.56***	58.89±11.32**	7.25±0.67	3.06±0.18	28.52±6.61

AmB = Amphotericin B at 5 mg/kg bw, CBD100 = *C*. *cassia* bark DCM fraction at 100 mg/kg bw, INF = infection control group, VC = vehicle control group,

^+++^ indicates *P*<0.001 with respect to normal group,

**P<0.01 and

*** *P*<0.01 in comparison to INF.

In hamster model, the adverse effects of CBD treatment (100 mg/kg bw) were assayed in normal and *L*. *donovani* infected hamsters. In case of uninfected animals, none of the hepatic and renal parameters swayed significantly from normal range in any of the treatment groups (Table 4). In case of infected animals, all the parameters were found to be in normal range except for ALP in all the experimental groups (Table 5). ALP levels were found to be notably enhanced (*P*<0.01) in infected and vehicle control animals. A significant decline in ALP activity was observed after CBD and AmB treatment (*P*<0.05). Both the data reinstated inertness of CBD towards renal and hepatic toxicity.

**Table 4:** Evaluation of hepatic and renal toxicity of CBD treatment in hamsters.

*In vivo* adverse effect in normal hamsters					
Groups	SGOT (U/L)	SGPT (U/L)	ALP (KA)	Creatinine (mg/dl)	Urea (mg/dl)
**Normal**	47.81±2.19	42.84±4.41	6.06±0.63	2.46±0.13	34.55±4.16
**AmB**	59.10±4.81	52.76±5.56	3.76±0.71	5.35±0.24	46.67±5.17
**CBD100**	52.06±5.07	49.05±6.76	3.18±0.83	4.14±0.36	39.86±3.16

**Table 5:** Assessment of hepatic and renal toxicity of CBD in *L*. *donovani* infected hamsters.

Effect on liver and kidney functions of infected hamsters					
Groups	SGOT (U/L)	SGPT (U/L)	ALP (KA)	Creatinine (mg/dl)	Urea (mg/dl)
**Normal**	36.51±2.22	49.08±2.23	7.51±1.64	1.44±0.089	29.76±4.25
**INF**	45.59±9.87	62.08±8.91	16.74±0.88^++^	1.73±0.079	40.57±2.4
**VC**	45.01±8.42	58.24±7.76	15.57±0.57^++^	1.63±0.084	38.92±1.79
**AmB**	38.86±2.65	51.78±5.49	9.36±0.86*	1.47±0.049	36.82±1.40
**CBD100**	41.96±7.99	55.57±6.69	8.65±2.48*	1.41±0.136	32.54±1.98

^++^ signifies *P*<0.001 with respect to normal group,

*P<0.05 in comparison to INF.

## Discussion

As a step towards VL elimination drive, the quest for better antileishmanials has become a necessity in the face of resurgence of apparently cured VL infections as PKDL, and its co-infection in AIDS patients. Also, in the absence of any effectual vaccine, the present chemotherapeutics are ineffective due to rapidly increasing drug resistance and toxicity [3, 4, 9]. Thus, switching to alternate therapies, especially plant-derived drugs may provide a breakthrough in the search for better, safe and cost-effective antileishmanials [33]. Use of plant extracts in case of leishmaniasis treatment has also been supported by WHO [34] and several studies in past have demonstrated potent leishmanicidal activities of plant extracts [33, 35]. Encouraged from the previous reports, and also harnessing the benefit of leishmaniasis from pre-purposed drugs [36, 37], we investigated antileishmanial potential of *Cinnamomum cassia* bark dichloromethane fraction against *L*. *donovani* parasites *in vitro* and *in vivo*. CBD exhibited discernable anti-promastigote efficacy and inhibited the growth of *L*. *donovani* promastigotes in time- and dose-dependent manner. Anti-promastigote effect of CBD was leishmanicidal in nature, which is preferred over leishmanistatic effect as it aids rapid parasite clearance thereby rendering lesser risk of relapse. Many plant fractions have been previously reported to be leishmanicidal in nature [33]. In fact, moderate antileishmanial activity of *C*. *cassia* essential oil has been reported by Le et al., where in the authors screened essential oils from different Vietnamese plants for activity against *L*. *mexicana* promastigotes [38]. The anti-promastigote activity of CBD was mediated via apoptosis as evidenced by PS externalization, mitochondrial membrane depolarization, ROS generation, DNA fragmentation and increased percentage of cells in sub G_0_/G_1_ population. Loss of mitochondrial function is critical to apoptosis induction and mitochondria are thought to be both source and target of ROS [39, 40]. Upon loss of Ψm, cells become committed to apoptosis and ROS has also been reported to participate in cellular DNA damage [41] which was characterized by TUNEL assay and cell cycle analysis. Aqueous extract of *C*. *cassia* bark has been divulged to induce apoptosis in human cancer cell line through loss of Ψm [42] indicating that *C*. *cassia* constituents are capable of initiating programmed cell death. Pentamidine, the known antileishmanial drug, used as positive control in this study has been regularly employed by us [24, 25] and others [43, 44] to compare the antileishmanial efficacy of different plant extracts or compounds. It was also employed as a positive control for all apoptotic studies as it is known to induce apoptosis in *Leishmania* promastigotes by inducing DNA fragmentation and by modulating mitochondrial function including Ψm depolarization [45, 46].

Since, amastigote form is the clinically relevant stage of *Leishmania* parasites, it is imperative for a drug to be able to inhibit the growth of *Leishmania* amastigotes. CBD also exhibited profound anti-amastigote efficacy, which was found to be independent of NO generation. Such NO independent leishmanicidal activity has also been previously observed by us [47] and others. We have earlier found that *P*. *nigrum* hexane and ethanolic fractions exhibited strong antileishmanial activity without significant NO production [47]. Some other leishmanicidal compounds such as piperine [48], trans-β-caryophyllene [49], nimodipine [50] and coronaridine [51] have also been demonstrated to exert their antileishmanial activity without inducing NO generation.

The plant secondary metabolites present in *C*. *cassia* DCM fraction that may have contributed to the observed leishmanicidal effect were identified by GC-MS analysis. Cinnamaldehyde detected in maximum proportion, is known to inhibit various cancer or tumor cell lines [52], and its antibacterial [53] and antifungal [54] properties are also reported. Cinnamaldehyde has also been observed to induce apoptosis in cancer cell lines [55] and may have contributed to apoptosis-inducing potential of CBD in our study. Another compound, o-methoxycinnamaldehyde, detected in CBD is reported to be antifungal [56]. Coumarin also detected in significant proportion is widely present in numerous plants and is endowed with multiple pharmacological properties. Coumarin, its various analogues and derivatives are reported to be antileishmanial [57, 58], antitrypanosomal [59], antifungal [60], anticancer [61] and antimicrobial [62]. Viridiflorol, another plant secondary metabolite present in CBD, has also been detected in other medicinal plant fractions or extracts that were reported to be antileishmanial [63], antifungal [64] and antibacterial [65].

The *in vivo* therapeutic potential of *C*. *cassia* bioactive fraction was evaluated both in BALB/c mice and hamster model of the disease. BALB/c mice are the most commonly used disease model as they are highly susceptible to *Leishmania* infection and also have been proven to be extremely useful for evaluation of mechanisms related to disease pathogenesis and cure [29, 66]. Hamsters are an optimal model to study the *in vivo* efficacy of potential antileishmanial formulations or compounds as hamsters closely mimic the clinical and immunopathological aspects of human disease [67]. Moderate levels of protection were achieved with CBD at 50 mg/ kg bw treatment whereas 100 mg/kg bw dose rendered significant protection in case of both *L*. *donovani* infected mice and hamsters. A small percentage of parasites still persisted in host liver and spleen, which has also been evident after successful treatment with other drugs [68, 69]. Also, it is very well established in VL that even after apparent cure, complete elimination of parasites is not achieved and despite this, recovered immune-competent hosts show all signs of clinical cure even after long periods of recuperation [70].

Since, recovery from *Leishmania* infection includes both direct parasite killing and an elevation of CMI, immunomodulatory nature of CBD treatment was investigated to determine its Th1 stimulatory potential. The positive control used for *in vivo* studies *i*.*e*., AmB in addition to being directly antileishmanial is also known to be Th1 inducing [71]. Therefore, AmB was also employed as a reference drug for all immunomodulation experiments. Cell-mediated immune responses were either only partially or marginally affected by CBD (100 mg/kg bw) treatment. DTH and lymphoproliferative responses were partially strengthened whereas NO production was negligibly stimulated in both mice and hamster models. This may be attributed to the presence of cinnamaldehyde (detected in notable amounts in CBD) which is known to favour the elicitation of anti-inflammatory immune response by decreasing the production of tumor necrosis factor-alpha (TNF-α), prostaglandin E2 (PGE2), Cyclooxygenase-2 (COX-2) [72]. Cinnamaldehyde has also been reported to suppress NO production by reducing the expression of nitric oxide synthase II (NOSII), an enzyme activated by Th1 cells that converts L-arginine into citrulline and NO. Another component, o-methoxycinnamaldehyde (or 2-methoxycinnamaldehyde) has been shown to inhibit IL-1β, a pro-inflammatory cytokine along with COX-2 activity [72, 73]. Both cinnamaldehyde and o-methoxycinnamaldehyde have been demonstrated to be anti-inflammatory as they inhibited NO generation and TNF-α production in RAW 264.7 and J774A.1 macrophages [74]. In fact, coumarin has also been reported to be associated with the induction of anti-inflammatory immune response [61]. Presence of these anti-inflammatory compounds may have led to restoration of only pro-inflammatory cytokines (INF-γ and IL-12) whereas IL-4 and IL-10 (anti-inflammatory cytokines) levels remained more or less unchanged in CBD treated BALB/c mice. Changes in serum levels of IgG2a and IgG1 are induced by INF-γ and IL-4, respectively, which in turn demonstrate the generation of Th1 or Th2 immune responses, respectively. There was neither significant decline in IgG1 nor appreciable elevation in IgG2a levels, which can also be corroborated with marginal changes in IL-4 and no potent stimulation of INF-γ levels with CBD.

Similar observations have been made with other plant extracts or plant-derived molecules. Chloroformic extracts of *Portulaca werdermannii* and *Portulaca hirsutissima* despite being antileishmanial had an inhibitory effect on lymphocyte proliferation [75]. Another compound Cyclosporin-A which is a known immunosuppressant with wide range of antiprotozoal activities, has been shown to inhibit *L*. *major* infection in NO and TNF-α independent manner. Further, its antileishmanial efficacy was not compromised in the presence of Th1 antagonizing cytokines such as IL-10 and IL-13 [76]. Sen et al. [77] reported *in vivo* efficacy of artemisinin, where artemisinin was found to be highly effective without inducing a Th1-biased immune response in *L*. *donovani* infected BALB/c mice. Artemisinin treatment was incapable of inducing NO production; it only restored the levels of pro-inflammatory cytokines to normal range and did not specifically alter IL-4 and IL-10 levels. All these studies suggest that even in the presence of mixed Th1/Th2 response, drugs can exhibit profound parasiticidal activity. Same has been advocated by Sen et al. [77], that therapeutic agents with direct parasiticidal action may even prove more beneficial when treating immunocompromised hosts.

The toxicity profile of *C*. *cassia* bioactive fraction was studied both *in vitro* and *in vivo*. CBD did not induce any cytotoxicity against peritoneal macrophages even at 500 μg ml^-1^ indicating its inertness. Cinnamaldehyde, the major constituent of CBD has been reported to be non-toxic against purified T cells and macrophages [52]. *C*. *cassia* is known to be hepatoprotective [78, 79] and in our studies also, CBD did not specifically alter serum levels of SGOT, SGPT and ALP from normal range in both normal mice as well as hamsters. Creatinine and urea levels also remained close to normal values in normal uninfected mice and hamsters. In *L*. *donovani* infected mice, SGOT and SGPT levels were found to be considerably enhanced whereas in infected hamsters, ALP levels were observed to be particularly elevated. All other parameters did not exhibit any significant deviation from normal range. CBD treatment brought down escalated SGOT and SGPT as well as ALP levels in infected mice and hamsters indicating its non-toxic nature.

Despite a plethora of knowledge about parasite biology and immunology, fight against leishmaniasis has been less fruitful than expected. Due to complex disease pathogenesis and interlinked immune-mechanisms, many encouraging leads are not being translated into clinical success. Thus, it is imperative to search for better antileishmanial drugs from alternate sources. In our present study, we investigated antileishmanial efficacy of *C*. *cassia* bark DCM fraction (CBD) and observed that the bioactive fraction bore a remarkable direct parasiticidal activity with partial modulation of Th1 immune response. We suggest, further exploration of the major plant secondary metabolites present in CBD for their antileishmanial efficacy and immunomodulatory potential that may shed insight into the mechanisms underlying their direct leishmanicidal capacity. Alternatively, the bioactive fraction may be standardized and used in combination with the known or pipeline drugs in synergy for effective maintenance of VL and as a stepping-stone in regional drive towards VL elimination.

## Supporting information

S1 Table Phytochemical profile of CBD as analyzed by GC-MS. (DOCX)
