# Supplementary material for: Cinnamomum cassia exhibits antileishmanial activity against Leishmania donovani infection in vitro and in vivo
Source: PLoS Negl Trop Dis. 2019 May 9;13(5):e0007227. doi: 10.1371/journal.pntd.0007227 (PMC6529017; doi:10.1371/journal.pntd.0007227)
Supplement: S1 Table — (DOCX) [file pntd.0007227.s001.docx]

**S1 Table Phytochemical profile of CBD as analysed by GC-MS**

| S. No. | RT | %Area | Compound |
| --- | --- | --- | --- |
|  | 10.708 | 0.06 | Burneol |
|  | 12.169 | 0.07 | 9-Methoxybicyclo[6.1.0]nona-2,4,6-triene |
|  | 12.299 | 0.42 | 3-Phenyl Acrolein |
|  | **14.163** | **36.27** | **Cinnamaldehyde** |
|  | 14.691 | 0.20 | Methyl(2E)-3-Phenyl-2-Propenoate |
|  | 15.247 | 0.28 | 2-Methoxycinnamic acid |
|  | 15.426 | 0.04 | 1-(Dimethoxymethyl)-3-Methoxybenzene |
|  | 16.036 | 0.14 | (+)-Cyclosativen |
|  | 16.039 | 0.92 | 1,2,4-Metheno-1H-Indene |
|  | 16.698 | 0.26 | Germacra-1(10),4(15),5-triene |
|  | 16.829 | 0.15 | (-)-Sinularene |
|  | **17.239** | **21.52** | **Cinnamaldehyde dimethyl acetal** |
|  | 17.469 | 0.08 | [1aR-(1a.α.4a.β,7.α,7a.β,7b.α)]-decahydro-1,1,7-trimethyl-4-methylene-1H-cycloprop[e]azulene |
|  | 17.750 | 0.02 | [S-(E,E)]-1-Methyl-5-methylene-8-(1-Methylethyl)-1,6-cyclodecadiene |
|  | 17.871 | 0.07 | 1-(3,3-Dimethyl-1-yl)-2,2-dimethylcyclopropene-3-carboxylic acid |
|  | 18.162 | 2.81 | Coumarin |
|  | 18.554 | 0.31 | (+)-Epi-bicyclosesquiphellandrene |
|  | 18.873 | 0.79 | γ-Murrolene |
|  | 19.120 | 0.10 | α-Guaiene |
|  | 19.267 | 0.11 | 1-Isopropyl-4-methyl-7-methylene-1,2,3,4,4a,5,6,7-octahydronaphthalene |
|  | 19.469 | 1.86 | α-Murrolene |
|  | 19.618 | 0.05 | β-Bisabolen |
|  | 19.780 | 0.23 | γ-Cadinene |
|  | 19.908 | 0.02 | 3,5-di-tert-butylphenol |
|  | 20.009 | 0.99 | 1,6,8-Trimethyl-1,2,3,4-tetrahydronaphthalene |
|  | 20.218 | 0.45 | Cadina-1,4-diene |
|  | **20.439** | **5.58** | **o-Methoxycinnamaldehyde** |
|  | 20.077 | 0.05 | 1,5-Epoxysalvial-4(14)-ene |
|  | 21.160 | 0.21 | Caryophyllenyl alcohol |
|  | 21.354 | 0.18 | Spathulenol |
|  | 21.487 | 0.42 | Elixene |
|  | 21.690 | 0.08 | Nerolidyl propionate |
|  | 21.800 | 0.02 | Illeudol |
|  | 21.887 | 0.10 | 1,5,5,8-Tetramethyl-3,7-cycloundecadiene-1-ol |
|  | 21.958 | 0.07 | Longifolenaldehyde |
|  | 22.037 | 0.35 | Myristaldehyde |
|  | 22.338 | 0.06 | 5-isopropyl-3,8-dimethyl-1,2-Naphthalenedione |
|  | 22.493 | 2.04 | Viridiflorol |
|  | 22.690 | 0.04 | 3,9-Dodecadiyne |
|  | 22.822 | 1.07 | α-Cadinol |
|  | 22.904 | 0.50 | δ-Cadinol |
|  | 23.098 | 0.25 | tau-Murrolol |
|  | 23.413 | 0.17 | Murolan-3,9(11) –diene-10-peroxy |
|  | 23.540 | 0.85 | Azulol |
|  | 23.755 | 0.09 | α-Bisabolol |
|  | 23.832 | 0.09 | Acetic acid, 3-(2,2-dimethyl-6-methylene-cyclohexylidene)-1-methyl-butyl ester |
|  | 23.917 | 0.10 | Acetic acid, 1-methyl-3-(2,2,6-trimethyl-bicyclo[4.1.0]hept-1-yl)-propenylester |
|  | 24.233 | 0.03 | 7-Hydroxydiacetoxyscripenol |
|  | 24.308 | 0.10 | 7-Hydroxymethyl-1,1,4a-trimethyl-6-methylenedecahydronaphthalen-2-ol |
|  | 24.511 | 0.11 | α,α,2,3,5,6-hexamethylbenzeneaceticacid |
|  | 24.748 | 0.13 | Cycloartanyl acetate |
|  | 24.927 | 0.23 | (-)-Globulol |
|  | 25.066 | 0.06 | 4-Isopropyl-1-methyl-2-cyclohexen-1-ol |
|  | 25.204 | 0.03 | (3S,4R,5R,6R)-4,5-Bis(hydroxymethyl)-3,6-dimethylcyclohexene |
|  | 25.408 | 0.13 | 9,19-Cyclolanostan-3-ol, acetate |
|  | 25.550 | 0.28 | 2-Hydroxyl-2,6-dimethyl-1-[3-methyl-1,3-butadienyl]bicycle[4.1.0]hept-3-yl acetate |
|  | 25.845 | 0.5 | 4-(2-Acetyl-5,5-dimethylcyclopent-2-enylidene)butan-2-one |
|  | 25.973 | 0.22 | (9Z)-9-Hexadecenal |
|  | 26.484 | 0.26 | 9,19-cyclo-9-β-lanostan-3β-ol acetate |
|  | 26.548 | 0.13 | 4,4,7a-Trimethyl-1,3-dimethyloctahydro-3ah-inden-3a-ol |
|  | 26.850 | 0.07 | Neophytadiene |
|  | 27.351 | 0.10 | 4,9-dihydroxy-6-methyl-3,10-dimethyl-3a,4,7,8,9,10,11,11a-octahydro-3H-cyclodeca[b]furan-2-one |
|  | 27.720 | 0.39 | Dihydrojasmone |
|  | 28.005 | 0.22 | 6-Isopropyl-3-methyl-2-cyclohexen-1-one |
|  | 28.508 | 0.03 | 14B-Pregnane |
|  | 28.626 | 0.30 | Methyl hexadecanoate |
|  | 29.500 | 0.13 | Palmitic acid |
|  | 29.760 | 0.15 | Vinyl palmitate |
|  | 30.996 | 0.03 | Carbonic acid, heptadecyl methyl ester |
|  | 31.180 | 0.04 | Oleyl alcohol trifluoroacetate |
|  | 31.410 | 0.05 | Bis (2,4-Ditert-butylphenyl) Pentanedioate |
|  | 31.686 | 0.08 | Z-2-Octadecen-1-ol acetate |
|  | 31.807 | 0.08 | Methyl (9E, 12E)-9,12-octadecanioate |
|  | 31.917 | 0.13 | Methyl eladiate |
|  | 32.437 | 0.12 | Triphenylamine |
|  | 32.530 | 0.04 | 5-Methyl-5-(4,8,12-trimethyltridecyl)dihydro-2(3H)-furanone |
|  | 32.972 | 0.11 | 2,3-Bis[9octadecenoyl]propyl(9E)-9-octadecenoate |
|  | 33.437 | 0.12 | Vinylsterate |
|  | 34.684 | 0.05 | N,N-Dimethylpalmitamide |
|  | 34.888 | 0.07 | Humulane-1,6-dien-3-ol |
|  | 35.334 | 0.08 | E-8-Methyl-9-tetradecen-1-ol acetate |
|  | 35.483 | 0.36 | 2-Methyl-3,4-diphenyl-2-cyclopenten-1-one |
|  | 35.850 | 0.06 | Methyl-9,10-dichlorooctadecanoate |
|  | 36.033 | 0.02 | Decane-2,5-dione |
|  | 36.099 | 0.02 | 4-Pentyl-1-(4-propylcyclohexyl)-1-cyclohexane |
|  | 36.302 | 0.03 | 5-Methyl-5-(4,8,12-trimethyltridecyl)dihydro-2(3H)-furanone |
|  | 38.638 | 0.10 | Dioctyl phthalate |
|  | 38.803 | 0.05 | 1(4-Isopropyl-Benzyl)-2(Naphthalen-1-yloxymethyl)-1H-Benzoimidazole |
|  | **39.516** | **10.92** | **1,2-Benzenediacrboxylic acid** |
|  | 39.807 | 0.03 | 5-Heptyl-2-hexyl-1-methyl-pyrrolidine |
|  | 40.819 | 0.03 | 4,4-dimethyl-5-nitro-3-methoxy-Benzo[b]dioxin |
|  | 41.194 | 0.03 | Dihydroaromadendrene |
|  | 41.596 | 0.05 | (4-Methyl-1-methylene-4-pentenyl)benzene |
|  | 41.948 | 0.03 | 1(22),7(16)-Diepoxy-tricyclo[20.8.0.0(7,16)]Triacontane |
|  | 42.508 | 0.23 | 1,2-Benzenedicarboxylic acid, diisodecyl ester |
|  | 42.946 | 0.55 | Diisodecyl phthalate |
|  | 43.092 | 0.06 | 1,2,3,5-Tetraisopropylcyclohexane |
|  | 43.233 | 0.04 | Trideuteriomethyl 10-epoxy-7-ethyl-3,11-dimethyltrideca-2,6-dienoate |
|  | 43.313 | 0.05 | 4,5-Diethyl-2,3-dimethyl-2,3-dihydrofuran |
|  | 43.781 | 0.10 | Methyl nonanoate |
|  | 43.947 | 0.03 | 2,6,9,2',6',6',9'-Octamethyl-[8,8']bi[tricyclo[5.4.0.0(2,9)]undecyl] |
|  | 44.140 | 0.04 | (3.β)-Stigmast-5-en-3-ol |
|  | 44.230 | 0.11 | 3-Cyclopentylpropinoic acid, 3-phenylpropyl ester |
|  | 44.536 | 0.11 | 22,23-Dibromostigmasterol acetate |
|  | 44.819 | 0.04 | Stigmasterol acetate |
|  | 44.997 | 0.04 | 3-Bromocholest-5-ene |
|  | 45.108 | 0.06 | 3β-Stigmasta-5,22-dien-3-ol acetate |
|  | 45.276 | 0.19 | Stigmast-5-en-3-ol oleate |
|  | 45.513 | 0.04 | α-Tocopherol |
|  | 46.329 | 0.12 | δ5-Ergosterol |
|  | 46.846 | 0.04 | Methyl melissate |
|  | 47.035 | 0.57 | γ-Sitosterol |
|  | 47.438 | 0.11 | Cholestenone |
|  | 47.903 | 0.05 | 3,5-Cholestadien-7-one |
|  | 48.137 | 0.05 | Stigmast-5-en-3-ol, oleate |
|  | 48.281 | 0.44 | Stigmast-4-en-3-one |
|  | 48.730 | 0.07 | Arteannuin b |
|  | 48.601 | 0.04 | 2-tert-Butyl-4,6-bis(3,5-di-tert-butyl-4-hydroxybenzyl)phenol |

RT=Retention time, major components are highlighted in bold
